# A Review on the Adsorption Isotherms and Design Calculations for the Optimization of Adsorbent Mass and Contact Time

**DOI:** 10.1021/acsomega.2c08155

**Published:** 2023-04-24

**Authors:** Orla P. Murphy, Mayank Vashishtha, Parimaladevi Palanisamy, K. Vasanth Kumar

**Affiliations:** † Department of Chemical Sciences, Synthesis and Solid State Pharmaceutical Research Centre and Bernal Research Institute, University of Limerick, Limerick, Ireland V94 T9PX; ‡ Department of Chemical and Process Engineering, Faculty of Engineering and Physical Sciences, School of Chemistry and Chemical Engineering, University of Surrey, Guildford, United Kingdom GU2 7XH

## Abstract

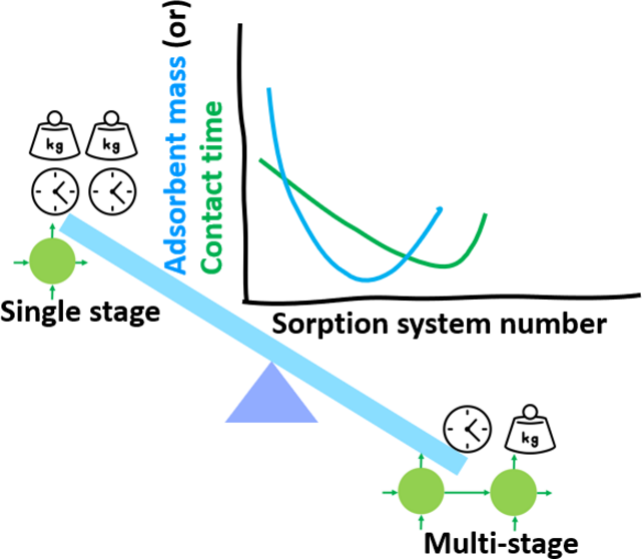

Adsorption is a widely used chemical engineering unit operation for the separation and purification of fluid streams. Typical uses of adsorption include the removal of targeted pollutants like antibiotics, dyes, heavy metals, and other small to large molecules from aqueous solutions or wastewater. To date several adsorbents that vary in terms of their physicochemical properties and costs have been tested for their efficacy to remove these pollutants from wastewater. Irrespective of the type of adsorbent, nature of the pollutant, or experimental conditions, the overall cost of adsorption depends directly on the adsorption contact time and the cost of the adsorbent materials. Thus, it is essential to minimize the amount of adsorbent and the contact time required. We carefully reviewed the attempts made by several researchers to minimize these two parameters using theoretical adsorption kinetics and isotherms. We also clearly explained the theoretical methods and the calculation procedures involved during the optimization of the adsorbent mass and the contact time. To complement the theoretical calculation procedures, we also made a detailed review on the theoretical adsorption isotherms that are commonly used to model experimental equilibrium data that can be used to optimize the adsorbent mass.

## 1 Introduction

Adsorption is an important chemical engineering unit operation and is widely used in the energy, environmental, and pharmaceutical sectors.^1^ In the environmental sector, adsorption is widely used to selectively remove various pollutants from wastewater.^2−10^ A few known examples include the recovery of heavy metals,^11^ pesticides,^8^ volatile organic compounds,^9^ dye molecules,^12^ antibiotics,^10,13^ and nitrates^3^ from wastewater. In the pharmaceutical industries, adsorption is commonly used to load drugs onto solid adsorbents for the controlled release of drugs.^14−16^ Irrespective of the application, type of fluid, target molecule to be adsorbed, or type of adsorbent, the efficacy of the adsorption process is primarily dictated by the potential of the adsorbent to selectively adsorb a specific target molecule from the bulk solution.^17^ On the other hand, the cost of the adsorption process directly depends on the cost of adsorbent and the contact time involved. To minimize the cost of the adsorption process, it is essential to minimize the cost of the adsorbent material. The common approach is to use low-cost adsorbents as an alternative to high-cost adsorbents.^18,19^ However, low-cost adsorbents often suffer from low adsorption capacity, which increases the mass of adsorbent required to remove a fixed percentage of solute, thus reducing the adsorption efficiency. (Note that adsorption efficiency can be defined as the capacity of a unit mass of adsorbent to remove a fixed percentage of solute from the solution or the capacity of a fixed mass of adsorbent to remove a fixed percentage of solute per unit time.) To reduce the cost while maintaining the high adsorption efficiency of a high-cost adsorbent, it is possible to develop experimental/design protocols that can minimize the mass of valuable absorbent materials and the contact time.^20−22^ Design methods that can minimize the adsorbent loading and the contact time, without affecting the adsorption efficiency, will be highly beneficial to the existing processes which heavily rely on high-cost but proven adsorbents like activated carbons and zeolites.

One method used by adsorption scientists to minimize the adsorption contact time and adsorbent loading without penalizing the adsorption efficiency is using a two-stage batch adsorption unit as opposed to a single-stage batch adsorption unit.^20−22^ In this review, we review the works that report on the optimization of the contact time and adsorbent loading using a two-stage batch adsorption unit. They report that the optimization of contact time and adsorbent loading relies on theoretical adsorption isotherms and kinetics. Furthermore, optimization of adsorbent mass or loading involves rigorous mathematical calculations that rely on theoretical adsorption kinetics and adsorption isotherms. A careful analysis of the adsorption literature shows that only a very few papers report on the design of a multistage adsorption unit for the optimization of contact time or adsorbent mass, probably due to the complex mathematical calculations involved. As mentioned above, optimization of the adsorbent mass or contact time requires rigorous mathematical calculations which require a strong mathematical background, which limits the usage of these established methods. To ensure that anyone who is interested in adsorption science but may not have a mathematical background can benefit from these established methods and apply these techniques in the future for different adsorption systems, we provide the step-by-step procedure for the optimization of the adsorbent mass or the contact time. Furthermore, we provide two solved examples (Example 1 and Example 2) that clearly explain the mathematical calculations involved and the procedures to minimize the contact time and the adsorbent loading. This paper is structured as a tutorial type of review to benefit researchers, especially adsorption scientists and analytical chemists who may not have a mathematical background. As the design of a multistage adsorption unit relies on the theoretical adsorption kinetics and isotherms, in this review, we list the different theoretical adsorption kinetics and isotherms that are widely used to represent the experimental adsorption kinetics or the equilibrium data.

This review is structured as follows:

Following the Introduction, the theoretical adsorption isotherms and kinetics are explained (section 2). These are commonly used to represent the adsorption kinetics and the equilibrium data as these isotherms are vital for the design calculations shown and reviewed in this work. In section 3, we focus on reducing the adsorbent loading. We show how the theoretical adsorption isotherms can be used to design a multistage adsorption unit to minimize the adsorbent loading as a function of percentage solute removal. In section 4, we show how to reduce the adsorption contact time for various design objectives by using the theoretical adsorption kinetics to design the multistage adsorption unit. In sections 5 and 6, we evaluate the works that report on the optimization of adsorbent mass and contact time, respectively, for different design objectives using a multistage batch adsorption unit. Finally, in section 7, we comment on the potential applications of the isotherms and kinetic expressions to design multistage adsorption systems for different targeted applications.

## 2 Theoretical Adsorption Isotherms and Kinetics

Multistage adsorption design calculations require a suitable theoretical expression that can predict the amount adsorbed, the solution concentration at equilibrium, and the amount adsorbed at any time as a function of the operating variables like temperature, initial concentration, and adsorbent mass. A suitable theoretical adsorption isotherm can help calculate the equilibrium concentration of solute in the solid phase and the liquid phase, whereas the amount adsorbed at any time can be obtained from any theoretical adsorption kinetics like the pseudo-first-order, pseudo-second-order, or diffusion-based models.

To optimize the adsorbent mass, it is essential to identify the theoretical isotherm that best fits the experimental equilibrium data. To optimize the contact time it, is essential to identify a suitable theoretical kinetic expression that best fits the experimentally obtained adsorption kinetic data.^20−22^ In Table 1 we list some of the commonly used and widely accepted theoretical adsorption isotherms, kinetics, and their linearized expressions and the way to obtain the isotherm or kinetic parameters involved in these expressions. In Table 1, for the convenience of the readers, we also explain the theoretical significance of these isotherms. From Table 1, it is evident that the isotherms and the kinetic parameters that contain two constants can be easily obtained using a linear regression analysis from their linear expressions. If the theoretical expression contains more than two parameters, then a nonlinear regression analysis can be used to obtain the isotherm parameters. For nonlinear regression analysis, researchers usually rely on iteration techniques, where the error distribution between the experimental data and the predicted isotherm or kinetics will be minimized by optimizing a suitable error function. (See the works reported in the literature.^23−27^) For demonstration purposes, in sections 3 and 4, we use only the Langmuir isotherm and pseudo-second-order kinetics to minimize the adsorbent loading and contact time, respectively. In these sections, the design objectives are defined and explained in detail and so are the procedures to optimize the contact time and the adsorbent loading using the theoretical adsorption kinetics and isotherms.

**Table 1:** Theoretical Adsorption Isotherms and Adsorption Kinetics and the Way to Obtain These Parameters^a^^,^^b^^,^^c^

model	nonlinear expression	linear expression	plot	isotherm parameters	theoretical significance^d^
Freundlich isotherm^28^	*q*_e_ = *K*_f_*C*_e_^*n*^	ln *q*_e_ = ln *K*_f_ + *n* ln *C*_e_	ln *q*_e_ vs ln *C*_e_	*K*_f_ = exp(intercept); *n* = slope	• This is an empirical expression and assumes that the adsorption site energies are exponentially distributed.
					• The parameter *n* can be related to the heterogeneity of the adsorbent surface. This value typically ranges from 0 to 1.
					• The relationship between *q*_e_ and *C*_e_ always follows a convex upward function, and thus the constant *n* is always less than unity.
					• At lower concentrations, *n* = 1, and thus this expression reduces to Henry’s expression.
					• This refers to heterogeneous adsorption of solute onto the adsorbent surface.
					• Nonideal and multilayer adsorption of solute onto the heterogeneous adsorbent surface.
					• This expression cannot predict the saturation limit of the adsorption isotherm.
					• If *C*_e_ and *q*_e_ are expressed in terms of mg/L and mg/g, then the units for the isotherm constant, *K*_f_, are (mg/g)/(L/mg)^*n*^.
Langmuir isotherm^29^	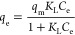	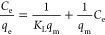	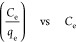	*q*_m_ = 1/slope; *K*_L_= slope/intercept	• This refers to homogenous adsorption of solute onto the adsorbent surface.
					• The heat of adsorption is the same for all the adsorption sites which can host the solute molecule, and this is independent of the already adsorbed molecules on the surface of the adsorbent.
					• Solute molecules are adsorbed onto the adsorption sites via chemisorption.
					• Solute–solute interactions between the adsorbed molecules are negligible, and once a molecule occupies the adsorption site, no further adsorption takes place. In that case, if the solute molecule hits the already adsorbed molecule, then it will be returned immediately to the bulk liquid. Theoretically, this leads to the conception of monolayer adsorption as the upper limit of the adsorption.
					• At low initial concentrations, this equation reduces to Henry’s law, and at higher concentrations, it predicts a monolayer adsorption capacity, *q*_m_.
					• Units: *C*_e_, mg/L; *q*_e_, mg/g; *q*_m_, mg/g; *K*_L_, L/mg
Redlich–Peterson^30^	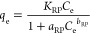	–	–	–	• This is an empirical expression that can excellently model the adsorption at lower and intermediate concentrations.
					• The isotherm can model both homogeneous and heterogeneous adsorption.
					• The constant *b*_RP_ ranges from 0 to 1. The value of *b*_RP_ = 1 indicates homogeneous adsorption, and *b*_RP_ < 1 indicates heterogeneous adsorption.
					• At higher concentration, this expression becomes *q*_e_ = (*K*_RP_/*a*_RP_)*C*_e_^1–*b*_RP_^.
					• At lower concentration, this expression reduces to Henry’s expression: *q*_e_ = *K*_RP_*C*_e_.
					• When *b*_RP_ = 1, this expression reduces to the Langmuir isotherm.
					• Experimental studies confirmed that this isotherm is a special case of the Langmuir isotherm when *b*_RP_ = 1 and is a special case of the Freundlich isotherm when *K*_RP_ and *a*_RP_ ≫ 1.^25,31−34^
					• Units: *C*_e_, mg/L; *q*_e_ mg/g; *a*_RP_, (L/mg)^*b*_RP_^; *K*_RP_, L/g
Sips (or) Langmuir–Freundlich^35,36^	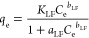	–	–	–	• This isotherm assumes theoretically there exist several sites on the adsorbent, each of which is characterized by a separate energy of adsorption.
					• The constant *b*_LF_ ranges from 0 to 1 depending on the level of adsorption heterogeneity. A value of *b*_LF_ < 1 indicates a heterogeneous adsorption.
					• When *b*_LF_ = 1, this expression reduces to the Langmuir isotherm or, in other words, a homogeneous adsorption.
					• At low concentration, *C*_e_ will be small and thus and this isotherm reduces to the Freundlich isotherm. In this case, the adsorption site energies will be exponentially distributed.
					• When *C*_e_ is large and *b*_LF_ = 1, the surface coverage approaches unity or a Langmuirian type of adsorption. In this case, the heats of adsorption of all the sites will be equal.
					• Units: *C*_e_, mg/L;, *q*_e_, mg/g; *K*_LF_, L/g; *a*_LF_, L/mg
Temkin^37,38^^e^	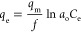	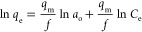			• This isotherm assumes the rates of adsorption and desorption increase exponentially with surface coverage.
					• The heat of adsorption and activation energy of adsorption vary exponentially with surface coverage.
					• The isotherm is not valid either for smaller or larger adsorption as it does not reduce to θ = 0 for *C*_e_ = 0 nor to θ = 1 for very large values of *C*_e_. This expression is valid only for the middle range of equilibrium concentrations.
					• Adsorption is heterogeneous.
					• This isotherm better predicts the adsorption on heterogeneous surfaces in the coverage region 0.2 < *q*/*q*_m_ < 0.8.
					• The heat of adsorption, the activation energy of adsorption, and desorption exhibit linear variations between the minimum and maximum values.
					• Units: *C*_e_, mg/L; *q*_e_, mg/g; *q*_m_, mg/g;, *a*_o_, L/mg; *f*, g/mg
Khan^39^	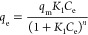				• This is an empirical expression with three isotherm constants, *q*_m_, *K*_1_, and *n*.
					• When *n* = 1, this expression reduces to the Langmuir isotherm with *q*_m_ equal to the maximum adsorption capacity and *K*_1_ characterizing the binding affinity of the adsorbent.
					• Note: In the original manuscript of Khan et al., it was shown that the *q*_m_ and *K*_1_ values obtained from this model were not comparable to the *q*_m_ and *K*_L_ values obtained using the Langmuir isotherm. Depending on the system, the *q*_m_ obtained using this isotherm was either higher or lower than the ones obtained from the Langmuir isotherm.
					• Units: *C*_e_, mg/L; *q*_e_, mg/g; *q*_m_, mg/g
Jossens^40^	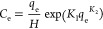	ln *C*e = ln *q*_e_ – ln *H* + *K*_1_*q*_e_^*K*_2_^			• This is an empirical expression with three isotherm constants, *K*_1_, *K*_2_, and *H*.
					• The constants *H* (L/g) and *K*_1_ [(mg/g)^−*K*_2_^] are functions of temperature given by the expressions and
					• The constants *C* and *K*_2_ (no units) are related to the distribution of the adsorption site energies.
Radke–Prausnitz^41^	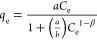	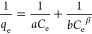			• This is a three-parameter empirical isotherm originally proposed by Radke and Prausnitz to represent the experimental equilibrium data of organic molecules from their dilute aqueous solutions over a wide range of concentration.
					• The parameter β is constrained to be less than unity.
					• At lower concentration, then this expression will reduce to Henry’s law of adsorption.
					• When the parameter β = 0, this isotherm reduces to a Langmuir isotherm,
					• At higher concentrations, ; then this expression will reduce to a Freundlich isotherm *q*_e_ = *bC*_e_^β^.
					• This isotherm can represent the experimental equilibrium data over a wide range of concentrations and adsorption occurring in at least three regions: Henry region (low concentration where the solute–solute interactions are almost negligible), intermediate concentration (Freundlich), and high pressure and near or complete saturation (Langmuir).
					• The binding site energies are quasi-Gaussian distributed skewed in the direction of high adsorption energies.^42^
					• Units: *C*_e_, mg/L; *q*_e_, mg/g; *a*, (mg/g)(L/mg)
Koble–Corrigan^43^	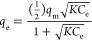	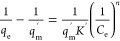			• This isotherm assumes the adsorption takes place as a chemical reaction between the “active centers” of the adsorbent and the adsorbate molecules being adsorbed. In other words, there is a dissociation of the adsorbate molecules.
	or 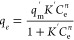				• In this expression *K* (L/mg) is an equilibrium constant and *q*_m_ (mg/g) is the maximum adsorption capacity.
					• For *n* = 1, this expression reduces to the Langmuir isotherm.
					• For low *C*_e_, *K*′*C*_e_^*n*^ ≪ 1; thus this expression reduces to Henry’s isotherm.
Frenkel–Halsey–Hill^44−46^	–ln(*C*_e_/*C*_s_) = *k*/(*q*_e_/*q*_m_)^*s*^ or *q*_e_ = *q*_m_{*k*/[−ln(*C*_e_/*C*_s_)]}^1/*s*^	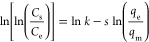			• At higher concentrations, the adsorption is dictated by both solute–sorbent and solute–solute interactions and the thickness of the adsorbed layer will be equal to several molecular layers.
					• This expression can explain the solidification (equivalent to condensation of multilayers of adsorbed gas molecules) of multilayers of the adsorbed molecules at a relatively larger distance from the surface. (Note: To our knowledge, this phenomenon was never experimentally verified during the adsorption of solute molecules from the liquid phase.)
					• The exponent *s* is based on the decay of the surface forces with distance. In other words, it describes the decay of the solute–sorbent interactions with distance.
					• For gas phase adsorption, the parameter *q*_m_ can be obtained from the Brunauer–Emmett–Teller isotherm. For liquid phase adsorption, while predicting isotherm using nonlinear regression analysis, the *q*_m_ value obtained from the Langmuir isotherm can be used as an initial guess value.
					• The above expression is flexible, and through simple modification of the constants *k* and *s*, it can be used to model any experimental equilibrium data that conform to type I, II or III shapes (see any standard textbook for the different types and shapes of adsorption isotherms).
Jura–Harkins isotherm^47−49^	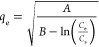	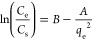			• Jura and Harkins proposed a new isotherm expression to model an S-shaped isotherm over a wide range of pressure that usually covers both monolayer and multilayered adsorption. This isotherm was proposed as an alternate to the commonly used BET isotherm and better fit the S-shaped isotherm or type II isotherms.
	(or) 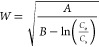	(or) 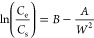			• The constant *A* is related to the surface area of the adsorbent and can be determined from the plot of ln(*C*_s_/*C*_e_) versus 1/*q*_e_^2^.
					• If a plot of ln(*C*_s_/*C*_e_) versus 1/*q*_e_^2^ is linear, then the adsorption is only due to monolayer adsorption.
					• In highly porous materials with larger pore volumes where we can expect a multilayered adsorption, a plot of ln(*C*_s_/*C*_e_) versus 1/*q*_e_^2^ will be curved.
					• An S-shaped isotherm is not frequently encountered in liquid phase adsorption as the concentrations of pollutants or target molecules in the solution phase are usually much less than the solubility of the target molecule in the solvent. Nevertheless, this isotherm can still capture the monolayer adsorption of the target molecule on any adsorbent surface.
					• The mass of adsorbate adsorbed onto unit mass of adsorbent, *q*_e_ (mg/g), can be expressed in volumetric units, *W* (cm^3^/g), using the expression *q*_e_ = *Wρ*_solute_, where ρ_solute_ (mg/cm^3^) is the solid density of the solute.
					• If the amount adsorbed, *W*, is expressed in terms of cm^3^/g, then the units for the isotherm constants *A* and *B* are cm^3^/g and g/cm^3^, respectively.
Unilan^50^	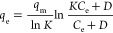				• The adsorbent surface is homotattic, and it contains different submicroscopic patches or regions of regular and uniform construction.
					• Each of these patches is a homogeneous surface and can be represented by a Langmuirian type of isotherm. Overall, the distribution of site energies of these individual homotattic patches is uniform.
					• The parameters *K* and *D* are isotherm constants.
					• Honig and Reyerson^50^ mentioned that this expression is the generalized form of the isotherm proposed by Temkin and Pyzhev (not cited here as we do not have access to this article).
Unilan^51,52^	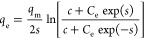				• There is a uniform distribution of adsorption energies among the surface sites.
					• The parameters *q*_m_, *c*, and *s* are isotherm constants. The parameter *c* characterizes the average adsorptive potential of the surface, and *s* characterizes the adsorption heterogeneity. The value of *s* will range from 0 to 1; a value of 1 indicates the surface is homogeneous.^52^
					• Units: *C*_e_, mg/L; *q*_e_, mg/g;, *q*_m_, mg/g; *c*, L/mg
Dubinin–Raduskevich^53,54^	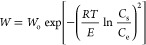				• The isotherm is also called the theory of volume filling of the micropores.
	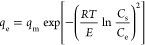				• The parameter *E* (J/mol) reflects the characteristic energy of the system, and it depends on the adsorbent and adsorbate.
					• We can write *E* = *βE*_0_, where β is a coefficient depending on the adsorbate.
					• The model assumes that there exists a fixed volume of micropores, *W*_0_ (cm^3^/g), that is filled to a capacity *W* (cm^3^/g) for any solute at a given value of *RT* ln(*C*_s_/*C*_e_)/β. The parameter *R* is a gas constant (J/mol·K), and *T* is the temperature in kelvin.
					• β is the affinity coefficient and is also called the similarity coefficient or relative molar works of adsorption. The β value for different solutes can be obtained from Polanyi’s relation which states that, at a fixed adsorption volume, [*RT* ln(*C*_s_/*C*_e_)]_1_/β_1_ = [*RT* ln(*C*_s_/*C*_e_)]_1_/β_1_ = [*RT* ln(*C*_s_/*C*_e_)]_reference_/β_reference_.
					• The β value of benzene is the standard reference (β(benzene) = 1). This parameter can also obtained from the molecular polarizabilities, *P*_e_, and molecular volume, *V*_m_, of adsorbate in the adsorbed state using the expression β = *P*_e_/*P*_e,reference_ = *V*/*V*_m,reference_. The values of *P*_e_ and *V*_m_ for a wide range of molecules can be found in the pioneering work of Wood.^55^
					• The volume of adsorbate filled in the pores available in the unit mass of adsorbent can be expressed in gravimetric units, *q*_e_ (mg/g), using the expression *q*_e_ = *Wρ*_solute_, where ρ_solute_ (mg/cm^3^) is the solid density of the solute. Likewise, the maximum adsorption capacity, *q*_m_, can be related to *W*_o_ by the expression *q*_m_ = *W*_o_ρ_solute_.
Tóth (expressions 1 and 2)^52,56,57^	expression 1: 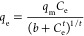				• Tóth obtained two different expressions to describe adsorption on both homogeneous and heterogeneous surfaces.
	expression 2: 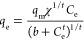				• This isotherm was developed based on the experimental observation that a heterogeneous surface is more adsorptive at the same pressure than a homogeneous adsorbent with constant binding energy for the adsorbate and with a specific surface area identical to that of the heterogeneous adsorbent.
					• The Tóth isotherm can predict the monolayer adsorption capacity of a homogeneous surface that will match the surface area predicted by the Langmuir isotherm.
					• When *t* = 1, the isotherm reduces to a Langmuirian type of isotherm and the adsorption energies of the binding sites will be uniformly distributed.
					• When *t* < 1, this isotherm can describe the heterogeneous adsorption. For lower values of *t* < 1 and for lower values of *C*_e_, the site energies will be exponentially distributed.^58^
					• As argued by Tóth, expression 2 is the thermodynamically correct expression.^52^
					• Units (expression 2): *C*_e_, mg/L;, *q*_e_, mg/g; *q*_m_, mg/g; χ, −; *b*, (mg/L)^*t*^
Jovanovic^59−62^	1 – θ = exp(−*aC*_e_) or *q* = *q*_*m*_(1 – exp(−*aC*_e_))	ln(*q*_m_ – *q*) = ln(*q*_m_) + *aC*_e_			• Theoretically, this isotherm defines the adsorption isotherm in terms of the uncovered part of the adsorbent surface, (1 – θ), and assumes this parameter decreases exponentially as a function of the equilibrium concentration.
					• The surface of the adsorbent is homogeneous but with a slightly different binding energy due to the periodicity of the crystal lattice which creates adsorption sites.
					• The parameter *q*_m_ is the adsorption capacity.
					• The constant *a* is related to the adsorption energy of the binding sites given by *a* = (1/*K*) exp(*E*/*RT*), where *K* is analogous to the isotherm constant, *K*_L_.
					• The adsorbed layer is mobile. The adsorbed molecules can hop from one adsorption site to another, and the energy barrier that hinders this molecular hopping movement is lower than that of desorption.
					• The binding site energies are quasi-Gaussian distributed skewed in the direction of high adsorption energies.
Vieth–Sladek^63^	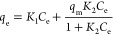				• The adsorption of solute at equilibrium is dictated by two components in the isotherm. The first one is described by a linear component using Henry’s law, and the second one is described by a nonlinear component based on a Langmuir isotherm.
					• In the original manuscript of Vieth and Sladek, in the linear region, *q*_e_ (mg/g) is assumed to increase proportionally with *C*_e_ (mg/L) and the proportionality constant was assumed to be equal to Henry’s parameter and a factor related to the amorphous volume fraction.
					• The constant *K*_1_ [(mg/g)(L/mg)] is Henry’s constant, *q*_m_ (mg/g) is the monolayer adsorption capacity, and *K*_2_ (L/mg) is a parameter related to the binding affinity of the adsorption sites for the solute molecule.
					• The linear components correspond to the adsorption on the surface, and the nonlinear component corresponds to the molecules adsorbed within the pore volume.
Fritz–Schluender^64^	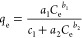			*a*_1_, *a*_2_, *b*_1_, *b*_2_, and *c*_1_ are isotherm constants.	• This is an empirical expression proposed mainly to target the best fit of multicomponent experimental adsorption equilibrium data, and this was done by introducing more than three isotherm parameters in the theoretical adsorption isotherm.
					• This expression can well represent the single component experimental adsorption equilibrium data.
					• This equation reduces into several well-known theoretical adsorption isotherms as special cases: (1) For *b*_1_ = *c*_1_ = *b*_2_ = 1, this expression reduces to the well-known Langmuir adsorption isotherm. (2) When *b*_1_ = *c*_1_ = 1, this equation reduces to the Redlich–Peterson isotherm. (3) When *c*_1_ = *a*_2_ = *b*_2_, the FS isotherm reduces to the Freundlich isotherm.
					• The binding site energies are quasi-Gaussian distributed skewed in the direction of high adsorption energies.^42^
pseudo-first-order kinetics^65^	*q*_*t*_ = *q*_e_(1 – e^–*K*_1_*t*^)	ln(*q*_e_ −*q*_*t*_) = ln *q*_e_ −*K*_1_*t*	ln(*q*_e_ – *q*_*t*_) vs *t*	*K*_1_= −slope; *q*_e_= exp(intercept)	• This is an empirical expression that can accurately predict the adsorption kinetics of the solute onto the adsorbent surface.
					• This assumes the adsorption kinetics at any instant of time is dictated by the concentration of the adsorption sites available on the adsorbent surface.
pseudo-second-order kinetics^20,66,67^	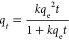	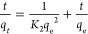	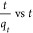	*q*_e_ = 1/slope; *k* = (slope)^2^/intercept	• This has monolayer adsorption.
					• The adsorption site energy is the same and independent of surface coverage.
					• Adsorption occurs only on the sites available on the surface, and there are no solute–solute interactions.
					• The adsorption rate is almost negligible when compared to that of the initial adsorption rate.

a The isotherm parameters in the nonlinear expressions can be obtained using a nonlinear regression analysis. Alternatively, isotherm parameters can be obtained from the slope and intercept of a linear plot generated according to the linear expressions shown in this table using a linear regression analysis technique.

b In the original manuscripts, the authors used different notations for the isotherm parameters. In this review for consistency or for convenience, we used different notations. It should be remembered that we cited the original articles for most of the isotherms reviewed in this table. For a few isotherms, we do not have access to the original articles and thus cited reliable sources and the works published by adsorption pioneers like Jaroniec, Rudzinski, Duong Do, Stoeckli, and Brunauer. Once the best-fit isotherm is identified, it can be used to expose the adsorption mechanism and nature of adsorption, and then it can be used to optimize the contact mass (which is discussed in section 3).

c Irrespective of the theoretical adsorption isotherms given in Table 1, the parameter *q*_m_ refers to the maximum adsorption capacity in the case of heterogeneous adsorption and it refers to the monolayer adsorption capacity in the case of homogeneous adsorption.

d Theoretical isotherms with more than two isotherm constants can be solved using a nonlinear regression analysis. The selection of best-fit isotherms using regression analysis and the error functions that are commonly used to predict the best-fit isotherm using nonlinear regression analysis is not reviewed in this work as it can be found elsewhere.^27,31,68−73^

e The mathematical derivation can be found in the works of Brunauer et al.^38^

## 3 Optimization of Adsorbent Mass

**Figure 1:**
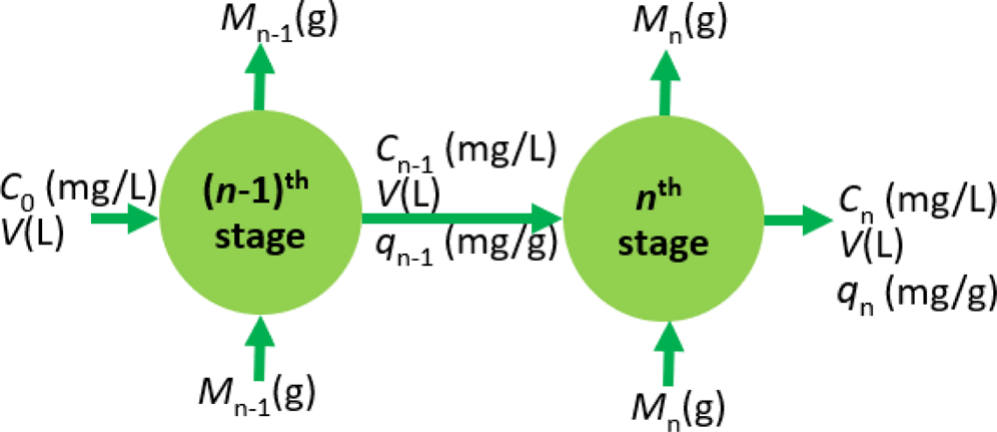
Schematic of the multistage adsorption unit.

To minimize the adsorbent mass, it is essential to perform batch adsorption in multiple stages. Therefore, it is essential to design a multistage adsorption unit.^74−76^Figure 1 shows the schematic of a typical multistage batch adsorption unit. In Figure 1, *C*_0_ is the initial concentration of the solute in the solution that enters the (*n* – 1)th adsorption stage, *C*_*n*–1_ is the equilibrium concentration of solute in the (*n* – 1)th stage, and *C*_*n*_ is the equilibrium concentration of solute in the *n*th stage. *M*_*n*–1_ refers to the required amount of adsorbent mass to bring the solute concentration from the initial concentration *C*_0_ to *C*_*n*–1_ at equilibrium conditions in the (*n* – 1)th stage. It is assumed that the volume *V* of the solution remains the same in all stages. Additionally, in each stage, we add fresh adsorbent to remove the solute from its bulk solution. In that case, *q*_0,*n*–1_ and *q*_0,*n*_ refer to the mass initial mass adsorbed at time *t* = 0 in the (*n* – 1)th and *n*th stages of the batch adsorber. As fresh adsorbent material is used in each stage *q*_0,*n*–1_ and *q*_0,*n*_ are equal to zero. Likewise, *M*_*n*_ refers to the mass of adsorbent required in the *n*th stage to bring the concentration of solution from *C*_*n*–1_ to *C*_*n*_ at equilibrium. The *q*_*n*–1_ and *q*_*n*_ in Figure 1 refers to the amount adsorbed at equilibrium in the (*n*–1)th and the *n*th stage, respectively.

Now, it is possible to perform the solute mass balance for the (*n* – 1)th stage of the multistage batch adsorption unit shown in Figure 1, as follows:
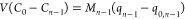
1

Likewise, the solute mass balance can be written for the *n*th stage as
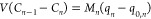
2

As mentioned earlier, fresh adsorbent is used at each stage, so eq 1 is rearranged to calculate the amount of solute adsorbed onto the unit mass of adsorbent for a desired amount of solute removal in the (*n* – 1)th stage using the reduced expression
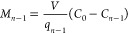
3

Similarly, for the *n*th stage, the amount of solute adsorbed onto the unit mass of adsorbent for a desired amount of solute removal can be obtained by rearranging eq 3 as follows:
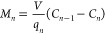
4

As we are using a fresh adsorbent in each stage, the amount adsorbed at equilibrium in each stage can be obtained from a suitable theoretical isotherm. If the experimental equilibrium data can be represented by a suitable theoretical adsorption isotherm, e.g., the Langmuir isotherm, then the amount adsorbed can be obtained using the Langmuir isotherm as follows:^29^
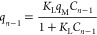
5and
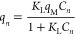
6Equations 5 and 6 can be substituted into eqs 3 and 4 to theoretically obtain the required amount of adsorbent in the (*n* – 1)th and *n*th stages, respectively. This can be altered for any initial concentration and for any desired solute removal from the solution (see eqs 7 and 8).

For the (*n* – 1)th stage
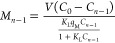
7

For the *n*th stage
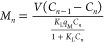
8

As shown in the model design calculations (see Example 1), eqs 7 and 8 require the adsorption isotherm parameters to calculate the required level of adsorbent mass in each adsorption stage of a multistage adsorption unit.

### 3.1 Example 1. Model Design Calculations to Optimize the Adsorbent Loading

Let us assume that we need to remove a pollutant from an aqueous solution using a suitable adsorbent at a fixed temperature *T*. The initial concentration of the solute in the solvent is 200 mg/L, and the volume of the solution to be treated is 60 L. Assume that we need to remove at least 95% of the pollutant from its aqueous solution using a two-stage sorption system. Assume that the experimental equilibrium for this model solute/adsorbent system follows a Langmuir isotherm and isotherm constants are given by *q*_m_ = 340 mg/g and *k*_L_ = 0.16 L/g. Design a two-stage sorption system that utilizes the minimum amount of adsorbent to remove 95% of the pollutant from the solution.

**Figure 2:**
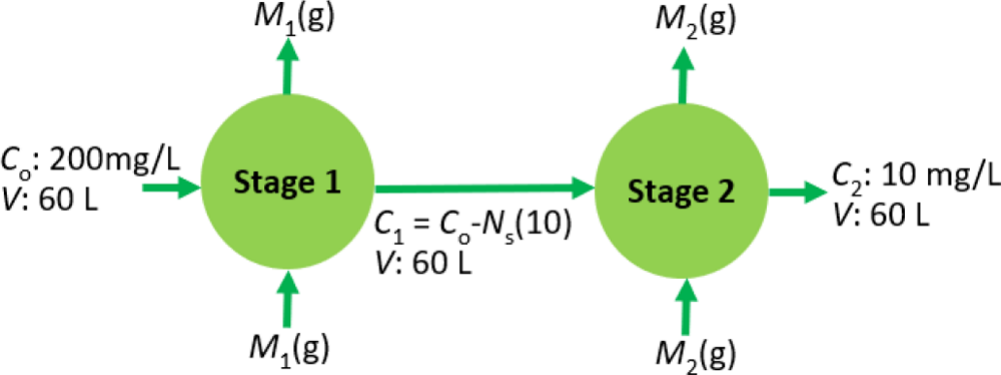
Schematic of a two-stage batch adsorption unit.

*Solution.* On the basis of the notations used in Figure 1, it is possible to graphically represent a two-stage adsorption unit as shown in Figure 2. *M*_1_ is the mass of the adsorbent required in stage 1 to bring the solution concentration from *C*_0_ to *C*_1_ at equilibrium. Likewise, *M*_2_ is the mass of adsorbent required in stage 2 to bring the solution concentration from *C*_1_ to the equilibrium solution concentration, *C*_2_. As the objective is to remove 95% of the solute from the solution, the equilibrium concentration of the solute in the liquid phase in the second stage is *C*_2_ = 10 mg/L.

To design the two-stage sorption system, it is essential to define the sorption system number. The sorption system number, *N*_s_, can be taken as a parameter that defines the equilibrium concentration of the solute in the liquid phase that exits stage 1. Based on the sorption system number, a series of equilibrium concentrations from 190 to 10 mg/L in 10 decrements will be considered in stage 1 of the two-stage sorption system. The relationship between the sorption system number and the assumed concentration of solute in the stage 1 of the two-stage adsorber is given in Figure 2. According to Figure 2, if the sorption system number *N*_s_ = 1, then the equilibrium concentration of the solute in the liquid phase in stage 1 will be equal to
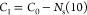
9
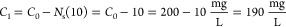
10

Likewise, if *N*_s_ = 6, then the equilibrium concentration of solute in stage 1 of the two-stage adsorber will be equal to
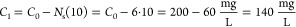
11

Finally, if *N*_s_ = 19, then the equilibrium solute concentration in the stage 1 will be equal to
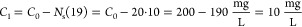
12

In other words, according to eq 9, based on the assumptions made for the design calculations, the value of *N*_s_ = 19 refers to a condition where a single-stage adsorption unit is enough to remove 95% of the initial solute concentration from the bulk of the liquid.

For the sorption system number 1, i.e., *N*_s_ = 1, it is possible to calculate the mass required to bring the solution concentration from *C*_0_ to *C*_1_ using eq 7:
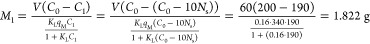
13

Similarly, for sorption system number 1, in stage 2, the mass of adsorbent required to decrease the concentration of solute in the liquid phase, *C*_1_ = 190 g/L, to *C*_2_ = 10 g/L at equilibrium can be calculated using eq 8 as follows:
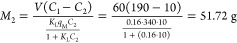
14

Thus, for the sorption system number *N*_s_ = 1, the total amount of adsorbent required to remove 95% of the solute from the solution of initial solute concentration *C*_0_ = 200 mg/L is equal to *M*_1_ + *M*_2_ = 1.822 + 51.72 = 53.44 g of adsorbent.

Likewise, for each sorption system number, it is possible to calculate the required amount of adsorbent to bring the solute concentration from *C*_0_ = 200 mg/L to *C*_2_ = 10 mg/L. For, example, if *N*_s_ = 6, the required amount of adsorbent mass in stage 1, *M*_1_, and in stage 2, *M*_2_, can be calculated as follows:
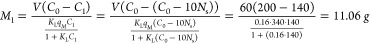
15
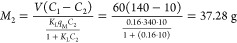
16

Thus, for the sorption system number *N*_s_ = 6, the total mass of adsorbent required to remove 95% of the solute from the solution is equal to *M*_1_ + *M*_2_ = 11.06 + 37.28 = 48.34 g.

**Figure 3:**
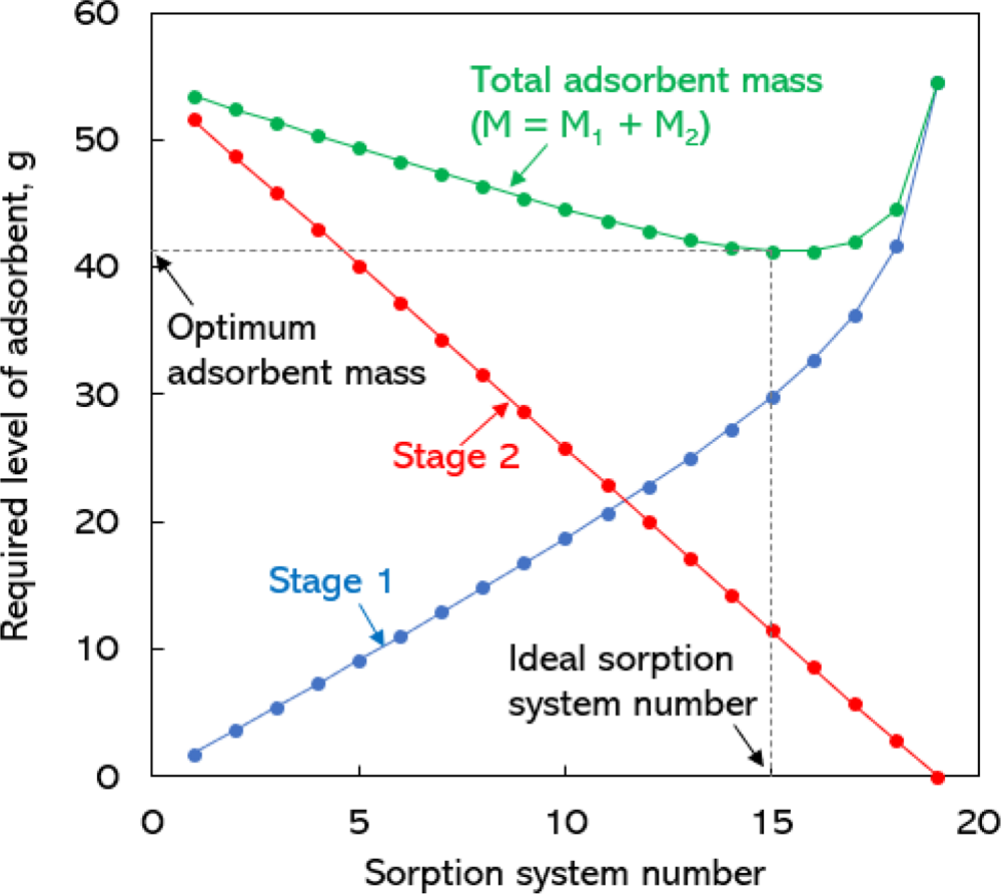
Plot of the required level of adsorbent mass in each stage versus the sorption system number for a fixed percentage removal of solute from a bulk solution of 60 L volume. Also shown is the total mass of the adsorbent used in all stages to remove a fixed percentage of solute. *C*_0_, 200 mg/L; *C*_2_, 10 mg/L; % solute to be removed, 95; number of adsorption stages, 2.

Following the model calculations shown above and using the expressions in eqs 10–14, it is possible to calculate the adsorbent mass required in each stage of the two-stage adsorber to remove a fixed percentage of solute from the bulk solution as a function of the sorption system number. If we know the amount of adsorbent used in each stage, then it is possible to calculate the total mass of the adsorbent required in both stages. This should equal the amount of adsorbent required to remove a fixed percentage of pollutant from the solution. In Figure 3, we show the plot of the amount the mass of adsorbent required in each stage of the two-stage adsorption unit as a function of the sorption system number. It is clear from the Figure 3 that, in stage 1, the required level of adsorbent to remove a fixed percentage of solute increases with an increase in *N*_s_. In stage 2, the mass of adsorbent decreases with a decrease in the sorption system number. From this we can find the optimum sorption system number and mass required to achieve our design objective of a fixed percentage removal of solute using a two-stage adsorption unit. The sorption system number *N*_s_ = 15, which requires the minimum amount of adsorbent to achieve the design target, can be called the ideal sorption system number. On the basis of our design calculations, we found that, when *N*_s_ = 15, a two-stage sorption system utilized the minimum amount of adsorbent, *M* = 41.25 g of adsorbent, to achieve the design target of 95% solute removal from the solution with an initial concentration of 200 mg/L. If we compare this value with the adsorbent mass required to achieve the same target of 95% solute removal with a single-stage adsorption unit (when *N*_s_ = 19), it can be realized that the two-stage adsorption unit requires 25% less adsorbent mass than the single-stage adsorption unit.

**Figure 4:**
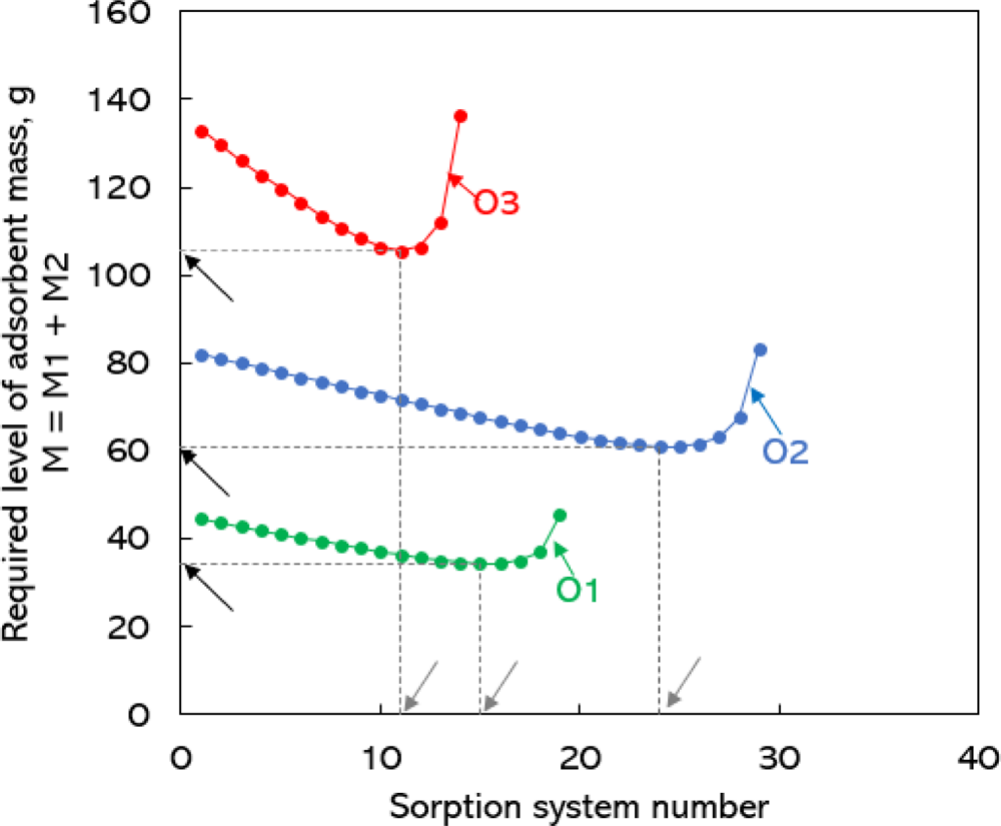
Required level of adsorbent mass in a two-stage adsorption unit versus the sorption system number for different targeted design objectives. O1, objective 1; O2, objective 2; O3, objective 3. See Table 2 for details of the design objectives. Black arrows indicate the optimum adsorbent mass, and the corresponding ideal sorption system number is shown using gray arrows.

In Figure 3, we show only the total adsorbent loading and the load of adsorbent in each of the stages of the two-stage batch adsorption unit to treat a solution of 60 L volume. Using the above explained method, it is possible to optimize the adsorbent loading to treat different volumes of solutions of different initial concentrations for any fixed percentage removal of solute by adsorption. In Figure 4, we show the optimum adsorbent loading and the ideal sorption system number for new design objectives listed in Table 2.

**Table 2:** Ideal Sorption System Number and the Minimum Adsorbent Loading Required in a Two-Stage Adsorption Unit to Achieve Three Different Design Objectives^a^

design objective	*V* (L)	optimum adsorbent loading (g)	ideal sorption system no.
O1: to decrease solution concentration from *C*_0_ = 200 mg/L to *C*_2_ = 50 mg/L	50	34.75	15
O2: to decrease solution concentration from *C*_0_ = 300 mg/L to *C*_2_ = 10 mg/L	60	61.04	24
O3: to decrease solution concentration from *C*_0_ = 150 mg/L to *C*_2_ = 10 mg/L	60	105.56	11

a Optimum adsorbent loading and the corresponding ideal sorption system number were obtained assuming that the relationship between *C*_1_, *C*_0_, and *N*_s_ follows eq 9.

## 4 Contact Time Optimization

**Figure 5:**
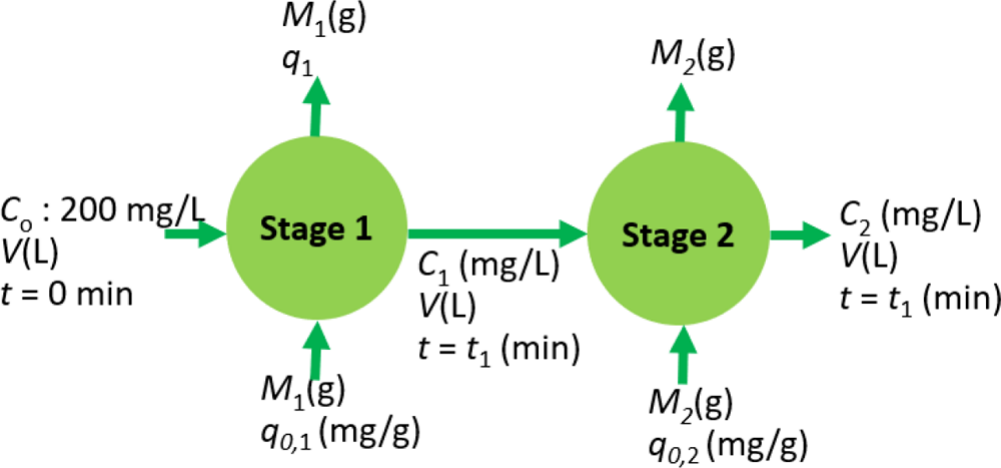
Schematic of the two-stage batch adsorption unit.

The batch adsorption contact time can be minimized without affecting the adsorption efficiency by performing the adsorption process using a multiple-stage adsorption unit.^20,77^ In Figure 5, we show the schematic of a two-stage batch adsorption unit. In Figure 5, *C*_0_ is the initial concentration of the solute in the solution that enters adsorption stage 1, *C*_1_ is the concentration of solute in the liquid that exits stage 1 at some instant of time *t*_1_, and *C*_2_ is the concentration of the solute that exits stage 2 at some instant of time *t*_2_. For simplicity, let us assume, in both stages 1 and 2, the volume and adsorbent loading are constant and are equal to *V* and *M*, respectively. Additionally, in each stage, we added fresh adsorbent to remove the solute from its bulk solution. The initial concentrations of the solute in the fresh adsorbent that is added to stage 1 and stage 2 of the two-stage adsorption unit are equal to *q*_0,1_ and *q*_0,2_, respectively. In that case, *q*_1_ and *q*_2_ refer to the masses of the solute adsorbed onto the unit mass of fresh adsorbent added in each stage of the two-stage batch adsorption unit. Furthermore, *q*_1_ and *q*_2_ refer to the solute concentrations in the solid phase in the stream that exits stage 1 and stage 2, respectively.

As we know the concentrations of solute in the solid and liquid phases, we can perform the solute mass balance for each stage of the two-stage batch adsorption unit shown in Figure 5, as follows:
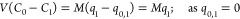
17

Likewise, for the second stage, the solute mass balance can be written as
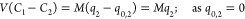
18

If the adsorption kinetics follows a pseudo-second-order kinetic expression, then the time required to achieve the concentration of solute in the liquid phase, *C*_1_, in stage 1 can be theoretically calculated by substituting the pseudo-second-order expression for *q*_1_ into eq 17 as follows:^20,22^
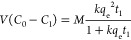
19

Similarly, the time required to achieve the concentration of solute in the liquid phase, *C*_2_, in stage 2 can be theoretically obtained by substituting the pseudo-second-order expression for *q*_2_ into eq 18 as follows:
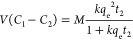
20

Using the above expressions, it is now possible to minimize the contact time required to achieve a fixed percentage of solute removal using a multistage sorption system (see the model calculation shown in Example 2).

### 4.1 Example 2. Model Calculation to Minimize the Adsorption Contact Time

Let us say we need to treat 1 L of solution that contains a pollutant (solute) of initial concentration *C*_0_ = 100 mg/L using the adsorption technique. Laboratory experiments confirmed that the adsorption kinetics follows a pseudo-second-order kinetics, and the theoretical parameters are found to be *k* = 0.003 g/mg·min and *q*_e_ = 112 mg/g. The design objective is to achieve a fixed percentage of 40% removal of pollutant in the minimum contact time.

*Solution.* As discussed above, to achieve a higher pollutant removal rate in less amount of time, it is essential to perform the removal of the pollutants using a multiple-stage adsorption unit. For convenience, we are using a two-stage adsorption unit (similar to the one shown in Figure 5). Furthermore, we assume that the volume and the mass of the adsorbent added to each stage of the two-stage adsorption unit are maintained a constant and are equal to 1 L (volume is given in the problem) and 0.5 g of adsorbent in each stage.

**Figure 6:**
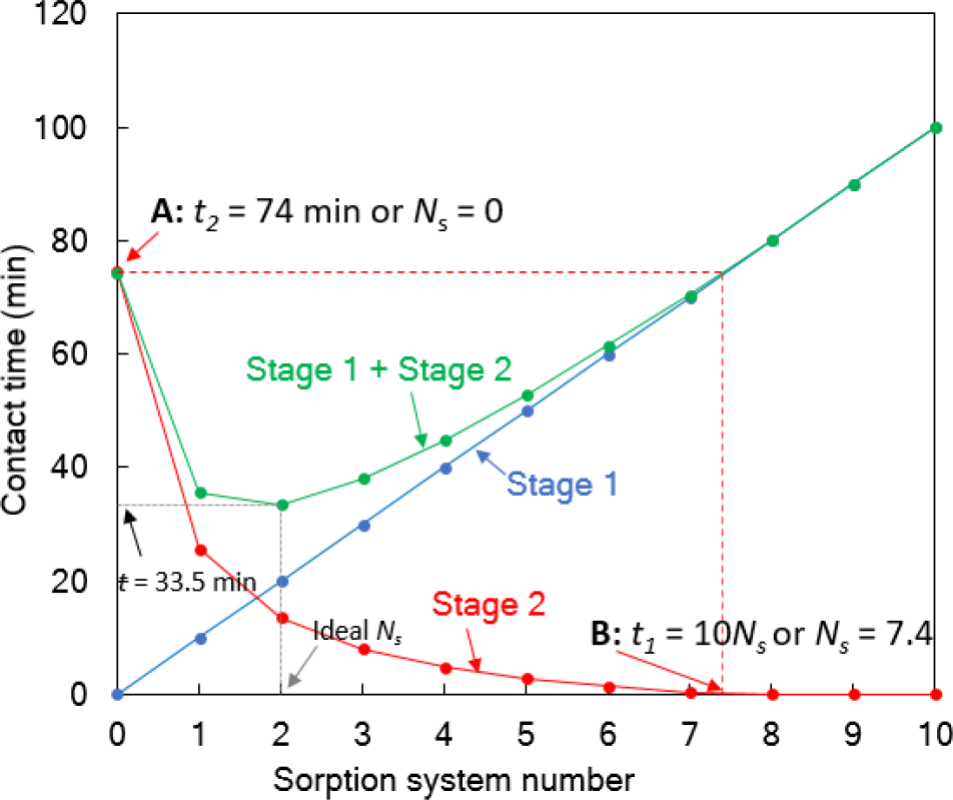
Plot of the adsorption contact time in each stage versus the sorption system number for a fixed percentage removal of solute from the bulk solution in a two-stage sorption unit. Also shown is the total contact time required in the two-stage sorption unit to remove a fixed percentage of solute. *V*, 1 L; M, 0.5 g; *C*_0_, 100 mg/L; *C*_2_, 60 mg/L; *t*_1_ = 10*N*_s_ min; % of solute to be removed, 40; number of adsorption stages, 2. Point A corresponds to the contact time required to remove 40% of solute using a single-stage adsorber (to be specific, stage 2). Point B corresponds to the sorption system number, where 40% removal of the solute is achieved using a single-stage adsorber (to be specific, stage 1).

The first step is to define a sorption system number, *N*_s_, that directly reflects the contact time of adsorption in stage 1. Here, the sorption system number, *N*_s_, can be taken as a parameter that defines the adsorption contact time between the solution and the adsorbent in stage 1. Based on the sorption system number, a series of adsorption contact times from 10 to 100 min in 10 min increments will be considered in stage 1 of the two-stage sorption system. The relationship between the sorption system number, *N*_s_, and the contact time in stage 1, *t*_1_, of the two-stage adsorber is given in Figure 6. According to Figure 6, we set a linear relationship between the sorption system number and the adsorption contact time in stage 1 of the two-stage batch adsorption unit. If the sorption system number *N*_s_ = 1, then the contact time between the solution and the adsorbent in the stage 1 will be equal to
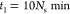
21

Similarly, if the sorption system number *N*_s_ = 10, then the contact time between the adsorbent and adsorbate in stage 1, *t*_1_, will be simply equal to 10·10 = 100 min.

For sorption system number 1, i.e., *N*_s_ = 1, it is possible to calculate the concentration of the solute in the liquid phase that exits stage 1 using eq 19 as follows:
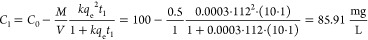
22

For stage 2, we set the initial concentration equal to the predicted final concentration of solute in the liquid phase that exits stage 1 of the two-stage batch adsorber. If the design objective is to remove a fixed percentage removal of solute from the solution using the two-stage batch adsorber, then it is possible to calculate the required amount of contact time in stage 2 to achieve that fixed percentage removal of solute using the analytical expression given by
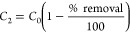
23

As our objective is to remove 40% of the initial solute concentration, *C*_0_ = 100 mg/L, that enters stage 1, the final concentration of the solute in the liquid phase that exits stage 2 can be obtained using eq 23 as follows:
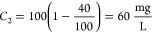
24

In stage 2, the target is to transfer the pollutant from the liquid to the solid adsorbent until the concentration of solute in the liquid phases reaches *C*_2_. The time *t*_2_ at which the solute concentration in the liquid phase reaches this final concentration, *C*_2_, can be theoretically calculated using the pseudo-second-order kinetic expression as shown earlier in eq 20.
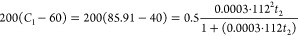
25

Equation 25 can be solved to predict *t*_2_ using a trial-and-error method (using the Goal Seek function available within Microsoft Excel), and *t*_2_ was found to be equal to 13 min. Thus, for sorption system number 1, the total contact time required to remove 40% of the solute from the solution of initial concentration, *C*_0_ = 100 mg/L, is equal to *t*_1_ + *t*_2_ = 10 + 13 = 33 min.

Following the model calculations explained above and using the expressions in eqs 17–20, it is possible to calculate the required contact time in each stage of the two-stage adsorber to remove a fixed percentage of solute from the bulk solution as a function of the sorption system number. If we know the required level of adsorption contact time in each stage, then it is possible to calculate the total amount of contact time required in both stages, which should be equal to the time required to remove a fixed percentage of pollutant (as per the design objective) from the solution. In Figure 6, we show the plot of the required level of contact time in each stage as a function of the sorption system number to achieve a fixed percentage (in this case 40%) removal of pollutant from the liquid phase. It is worthwhile to mention here that when *N*_s_ = 0 then, according to eq 19, *t*_1_ = 0. In that case, *C*_1_ = *C*_0_ and thus the contact time (see point A in Figure 6) required to remove the desired percentage of solute will simply reflect the time required to achieve the design objective using a single-stage adsorption unit. (Note that when *N*_s_ = 0 the entire solute removal will be achieved in stage 2.) Figure 6 clearly shows that, in stage 1, as expected the required level of contact time to remove a fixed percentage of solute increases with an increase in the sorption system number, *N*_s_, following eq 21. In stage 2, the contact time decreases with an increase in the sorption system number. Thus, the total time, which is the sum of *t*_1_ + *t*_2_, decreases with an increase in the sorption system number, *N*_s_, reaching a minimum or an optimum contact time when *N*_s_ is equal to the ideal sorption system number (in this case, *N*_s_ ∼ 1.8; see Figure 6). After that, the contact time increases with a further increase in the sorption system number until *N*_s_ ∼ 7.4.

As mentioned above, point A in Figure 6 theoretically represents that the calculated time required for a fixed percentage (40%) removal of solute in a single stage is 74 min (i.e., *t* = *t*_2_ = 74 min as *t*_1_ = 0 min). This means if *N*_s_ ≥ 7.4 (point B), theoretically we have already reached the desired objective of 40% solute removal in stage 1 of the two-stage batch adsorber. This can be visually observed in Figure 6, where the contact times *t*_2_ = 74 min for *N*_s_ = 0 and *t*_1_ = 74 min when *N*_s_ = 7.4 intersect (see the dashed red lines in Figure 6). This means *N*_s_ = 0 and *N*_s_ = 7.4 refer to the theoretical fact that only one stage of the two-stage adsorber is sufficient to achieve the design target of 40% pollutant removal from the solution. It is clear from Figure 6 that, when *N*_s_ = 7.4 and *N*_s_ = 0, the contact time required to remove the 40% of the solute is roughly 2.4 times higher than the optimized contact time (∼33.5 min; see Figure 6) using a two-stage adsorption unit. The sorption system number at which the total contact time reaches the optimum can be taken as the ideal sorption system number. Through the design calculations, we found that, when *N*_s_ ∼ 1.8, a two-stage sorption system requires the least amount of contact time to achieve the design target of 40% solute removal from the solution with an initial concentration of 100 mg/L.

**Figure 7:**
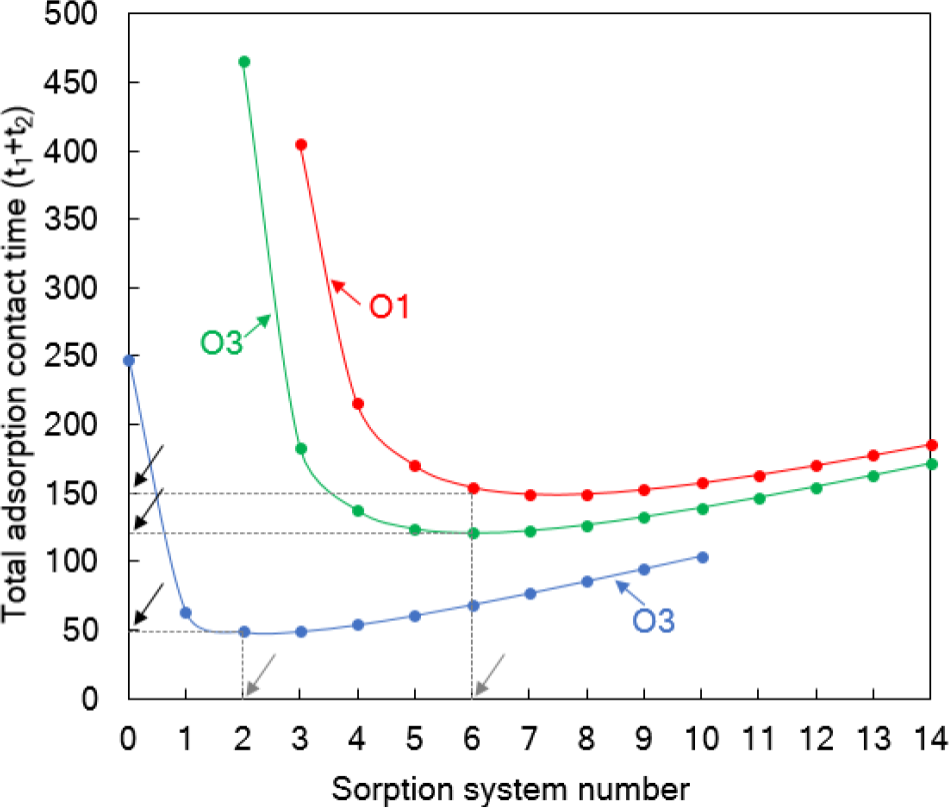
Adsorption contact required in each stage of the two-stage adsorption unit versus the sorption system number for different design objectives. O1, objective 1; O2, objective 2; O3, objective 3. See Table 3 for details of the design objectives. Black arrows indicate the optimum or minimum contact time to achieve a fixed percentage removal of solute from the bulk solution, and the gray arrows represent the corresponding ideal sorption system number.

Using the design protocols described above, it is possible to optimize the contact time to treat different volumes of solutions of different initial concentrations, different adsorbent loadings, and different temperatures for any fixed percentage removal of solute by adsorption. In these cases, it is essential to develop a relationship between the pseudo-second-order kinetic constant, *k*, and the theoretical sorption capacity, *q*_e_, as a function of the operating variables like adsorbent mass, initial concentration, and temperature. For demonstration purposes, we optimized the contact time for three different design objectives listed in Table 3 and the results are shown in Figure 7. In Table 3, we also provide the *k* and *q*_e_ values used to optimize the contact time for the listed design objectives. From Figure 7 and Table 3, it can be seen that the design protocol explained so far is flexible and allows optimization of the contact time as a function of initial concentration, solution volume, and mass of adsorbent for any fixed percentage removal.

**Table 3:** Minimum Contact Time and the Corresponding Ideal Sorption System Number for Three Different Design Objectives

design objective	*V* (L)	*M* (g)	*k* (g/mg·min)	*q*_e_ (mg/g)	optimum contact time (min)	ideal sorption system no.
O1: to minimize the contact time for 40% removal of solute from solution of initial concentration *C*_0_ = 100 mg/L	2	0.5	0.0003	112	149	7
O2: to minimize the contact time for 50% removal of solute from solution of initial concentration *C*_0_ = 100 mg/L	1	0.5	0.0003	112	48	2
O3: to minimize the contact time for 40% removal of solute from solution of initial concentration *C*_0_ = 50 mg/L	3	0.5	0.0003	112	120	6

## 5 Optimization of Adsorbent Mass Using Theoretical Adsorption Isotherms: Review

In Table 4, the works available in the literature that report on the design of multistage adsorption units to optimize the adsorbent loading using different theoretical adsorption isotherms are listed. Table 4 also lists the adsorption isotherms used and the isotherm constants of the different adsorption systems reviewed in this work. For the purpose of a comparison, for some of the systems reported in the literature, we show the adsorbent loadings in the first and second stages and the total adsorbent loading during the removal of some fixed percentage of the targeted pollutants using a two-stage adsorption unit. For all the different combinations of the adsorbents and adsorbates listed in Table 4, we presented the ratio of the adsorbent loading in the multistage adsorption unit to the adsorbent loading in the single-stage adsorption unit (*M*/(*M*_1_ + *M*_2_) for a fixed percentage removal of a targeted pollutant. For a fixed percentage removal, and for the assumed sorption system number, if this ratio is equal to 2, then it signifies the fact that the total adsorbent loading can be reduced by half while using a two-stage adsorption unit instead of a single-stage adsorption unit. For a few adsorbent/adsorbate systems, we noticed that, for a fixed percentage removal of a specific adsorbate, a two-stage adsorption system minimizes the adsorbent loading roughly from ∼3 to 5 times. In general, the lower the initial concentration, the higher is the ratio of the adsorbent loading in a two-stage adsorption unit to that in a single-stage adsorption unit. In some extreme case, for a fixed percentage removal of solute, the adsorbent loading was roughly 18 times lower while using a two-stage sorption unit than the amount of adsorbent mass required in a single-stage adsorption unit. The adsorbents/adsorbates listed in Table 4 differ from each other in terms of their physicochemical properties. From Table 4 it can be seen that only a handful of theoretical adsorption isotherms, including Freundlich,^28^ Langmuir,^29^ Langmuir–Freundlich,^35,36^ and Redlich–Peterson,^30^ are frequently used to optimize the adsorbent mass. This is due to their capability to represent the experimental adsorption equilibria of many combinations of adsorbents and adsorbates (irrespective of their physicochemical properties) at different temperatures. Another possible reason is that these expressions contain only two to three isotherm parameters which can be easily predicted or solved using a simple linear or nonlinear regression analysis. The key observation from Table 4 is that, irrespective of the nature and the type of the adsorbent or the adsorbate, during batch adsorption, removing the adsorbate in two stages effectively minimizes the adsorbent loading, especially while dealing with solution that contains a lower amount of adsorbate concentration.

**Table 4:** Adsorbent Mass Optimized Using Different Theoretical Adsorption Isotherms for a Wide Range of Adsorbents/Adsorbates^a^^,^^b^

adsorbate	adsorbent	% removal	isotherm/parameters	*C*_0_^c^ (mg/L)	*V* (L)	*M*_1_ (g)	*M*_2_ (g)	*M*_1_ + *M*_2_ (g)	*M*^d^ (g)	*M*/(*M*_1_ + *M*_2_)^d^	ref
malachite green	phosphoric acid activated date pits	99.5	Langmuir–Freundlich isotherm: *K*_LF_ = 14.8 L/g, *a*_LF_ = 0.221 L/mg, *b*_LF_ = 0.840; *C*_e_: mg/L and *q*_e_: mg/g	50	1	–	–	2.5	11.5 (11.5)	(4.6)	(78)
malachite green	phosphoric acid activated date pits	99.5		100	1	–	–	3.5	13.5 (13.5)	(3.86)	
malachite green	phosphoric acid activated date pits	99.5		150	1	–	–	4.4	15.1 (15.1)	(3.43)	
malachite green	phosphoric acid activated date pits	99.5		200	1	–	–	5.2	16.4 (16.4)	(3.15)	
malachite green	phosphoric acid activated date pits	99.5		250	1	–	–	6	17.6 (17.6)	(2.93)	
malachite green	phosphoric acid activated date pits	99.5		300	1	–	–	6.8	18.8 (18.8)	(2.76)	
malachite green	phosphoric acid activated date pits	99.5		350	1	–	–	7.6	19.9 (19.9)	(2.62)	
malachite green	phosphoric acid activated date pits	99.5		400	1	–	–	8.4	21 (21)	(2.5)	
malachite green	phosphoric acid activated date pits	99.5		450	1	–	–	9.2	22 (21.9)	(2.39)	
malachite green	phosphoric acid activated date pits	99.5		500	1	–	–	10	23 (22.9)	(2.3)	
malachite green	phosphoric acid activated date pits	95		50	1	–	–	1.3	2.2 (2.19)	(1.69)	
malachite green	phosphoric acid activated date pits	95		100	1	–	–	2.1	3.1 (3.07)	(1.48)	
malachite green	phosphoric acid activated date pits	95		150	1	–	–	2.9	3.9 (3.9)	(1.34)	
malachite green	phosphoric acid activated date pits	95		200	1	–	–	3.6	4.7 (4.69)	(1.31)	
malachite green	phosphoric acid activated date pits	95		250	1	–	–	4.3	5.5 (5.46)	(1.28)	
malachite green	phosphoric acid activated date pits	95		300	1	–	–	5	6.2 (6.23)	(1.24)	
malachite green	phosphoric acid activated date pits	95		350	1	–	–	5.8	7 (6.9)	(1.21)	
malachite green	phosphoric acid activated date pits	95		400	1	–	–	6.5	7.7 (7.74)	(1.18)	
malachite green	phosphoric acid activated date pits	95		450	1	–	–	7.3	8.5 (8.49)	(1.16)	
malachite green	phosphoric acid activated date pits	95		500	1	–	–	8	9.2 (9.24)	(1.15)	
malachite green	coconut shell activated carbon	99	Langmuir isotherm: *q*_m_ = 70.8 mg/g, *K*_L_= 1.64 L/mg; *C*_e_: mg/L and *q*_e_: mg/g	50	0.1	0.065	0.0128	0.0777	(0.16)	(2.06)	(79)
malachite green	thermally activated date stones	99.5	Langmuir–Freundlich isotherm: *K*_LF_ = 11.47 L/g, *a*_LF_ = 0.073 L/mg, *b*_LF_ = 0.341; *C*_e_: mg/L and *q*_e_: mg/g	50	1	2.21	0.981	3.192	(7.28)	(2.28)	(80)
malachite green	thermally activated date stones	99.5		100	1	3.6	1.567	5.173	(11.6)	(2.25)	
malachite green	thermally activated date stones	99.5		125	1	4.23	1.823	6.051	(13.5)	(2.23)	
malachite green	thermally activated date stones	99.5		150	1	4.82	2.064	6.882	(15.3)	(2.22)	
malachite green	thermally activated date stones	99.5		175	1	5.38	2.292	7.674	(17)	(2.22)	
malachite green	thermally activated date stones	99.5		200	1	5.92	2.511	8.437	(18.6)	(2.21)	
malachite green	thermally activated date stones	99.5		225	1	6.45	2.721	9.174	(20.2)	(2.2)	
malachite green	thermally activated date stones	99.5		250	1	6.96	2.924	9.89	(21.7)	(2.19)	
malachite green	thermally activated date stones	99.5		300	1	7.95	3.313	11.267	(24.6)	(2.18)	
malachite green	thermally activated date stones	99.5		325	1	8.43	3.5	11.933	(25.9)	(2.17)	
malachite green	thermally activated date stones	99.5		350	1	8.90	3.683	12.586	(27.3)	(2.17)	
malachite green	thermally activated date stones	99.5		400	1	9.82	4.037	13.857	(29.9)	(2.16)	
malachite green	thermally activated date stones	99.5		450	1	10.71	4.378	15.088	(32.4)	(2.15)	
malachite green	thermally activated date stones	99.5		500	1	11.58	4.708	16.288	(34.9)	(2.14)	
malachite green	microwave activated date stones	99.5	Redlich–Peterson isotherm: *K*_RF_ = 160.9 L/g, *a*_RF_ = 2.545 L/mg, *b*_RF_ = 0.927; *C*_e_: mg/L and *q*_e_: mg/g	50	1	0.72	0.171	0.888	(2.1)	(2.36)	(80)
malachite green	microwave activated date stones	99.5		100	1	1.27	0.268	1.535	(2.9)	(1.88)	
malachite green	microwave activated date stones	99.5		125	1	1.53	0.32	1.848	(3.3)	(1.77)	
malachite green	microwave activated date stones	99.5		150	1	1.78	0.374	2.156	(3.6)	(1.69)	
malachite green	microwave activated date stones	99.5		175	1	2.03	0.428	2.459	(4.0)	(1.63)	
malachite green	microwave activated date stones	99.5		200	1	2.28	0.484	2.759	(4.4)	(1.59)	
malachite green	microwave activated date stones	99.5		225	1	2.51	0.541	3.056	(4.7)	(1.55)	
malachite green	microwave activated date stones	99.5		250	1	2.75	0.598	3.35	(5.1)	(1.52)	
malachite green	microwave activated date stones	99.5		300	1	3.21	0.714	3.931	(5.8)	(1.48)	
malachite green	microwave activated date stones	99.5		325	1	3.44	0.773	4.219	(6.2)	(1.46)	
malachite green	microwave activated date stones	99.5		350	1	3.67	0.832	4.505	(6.5)	(1.45)	
malachite green	microwave activated date stones	99.5		400	1	4.12	0.95	5.071	(7.2)	(1.42)	
malachite green	microwave activated date stones	99.5		450	1	4.56	1.07	5.633	(7.9)	(1.4)	
malachite green	microwave activated date stones	99.5		500	1	5	1.19	6.19	(8.6)	(1.39)	
Allura direct red	treated peanut hull waste	99	Langmuir–Freundlich isotherm: *K*_LF_ = 17.48352 L/g, *a*_LF_ = 0.00628 L/mg, *b*_LF_ = 0.2929; *C*_e_: mg/L and *q*_e_: mg/g	10	0.5	0.02	0.0096	0.0300	(0.56)	(18.67)	(76)
Allura direct red	treated peanut hull waste	99		20	0.5	0.0325	0.0157	0.0500	(0.9)	(18.2)	
Allura direct red	treated peanut hull waste	99		30	0.5	0.0434	0.0209	0.0600	(1.2)	(20.17)	
Allura direct red	treated peanut hull waste	99		40	0.5	0.0531	0.0255	0.0800	(1.5)	(18.63)	
Allura direct red	treated peanut hull waste	99		50	0.5	0.0623	0.0300	0.0900	(1.7)	(19.33)	
Allura direct red	treated peanut hull waste	99		60	0.5	0.0709	0.0341	0.1100	(1.9)	(18)	
Allura direct red	treated peanut hull waste	99		70	0.5	0.0791	0.0380	0.1200	(2.2)	(18.42)	
Allura direct red	treated peanut hull waste	99		80	0.5	0.0870	0.0418	0.1300	(2.4)	(18.69)	
Allura direct red	treated peanut hull waste	95		10	0.5	0.0147	0.0087	0.0200	(0.3)	(16.5)	
Allura direct red	treated peanut hull waste	95		20	0.5	0.0240	0.0143	0.0400	(0.5)	(13.5)	
Allura direct red	treated peanut hull waste	95		30	0.5	0.0320	0.0191	0.0500	(0.7)	(14.6)	
Allura direct red	treated peanut hull waste	95		40	0.5	0.0392	0.0233	0.0600	(0.89)	(14.83)	
Allura direct red	treated peanut hull waste	95		50	0.5	0.0460	0.0274	0.0700	(1.04)	(14.86)	
Allura direct red	treated peanut hull waste	95		60	0.5	0.0524	0.0312	0.0800	(1.2)	(15)	
Allura direct red	treated peanut hull waste	95		70	0.5	0.0585	0.0348	0.0900	(1.33)	(14.78)	
Allura direct red	treated peanut hull waste	95		80	0.5	0.0643	0.0383	0.1000	(1.46)	(14.6)	
Allura direct red	treated peanut hull waste	90		10	0.5	0.0121	0.0080	0.0200	(0.26)	(13)	
Allura direct red	treated peanut hull waste	90		20	0.5	0.0198	0.0131	0.0300	(0.42)	(14)	
Allura direct red	treated peanut hull waste	90		30	0.5	0.0264	0.0175	0.0400	(0.56)	(14)	
Allura direct red	treated peanut hull waste	90		40	0.5	0.032	0.0214	0.0500	(0.69)	(13.8)	
Allura direct red	treated peanut hull waste	90		50	0.5	0.038	0.0252	0.0600	(0.81)	(13.5)	
Allura direct red	treated peanut hull waste	90		60	0.5	0.043	0.0287	0.0700	(0.92)	(13.14)	
Allura direct red	treated peanut hull waste	90		70	0.5	0.048	0.0320	0.0800	(1.03)	12.88	
Allura direct red	treated peanut hull waste	90		80	0.5	0.053	0.0352	0.0900	(1.13)	(12.56)	
reactive yellow K-4G	EPIDMA-bentonite^e^	85	Freundlich isotherm: *K*_F_ = 18.48 [(mg/g)(L/mg)], *n* = 0.167; *C*_e_: mg/L and *q*_e_: mg/g	50	10000	9000	5640	14640	16430 (16427)	(1.12)	(81)
reactive yellow K-4G	EPIDMA-bentonite	90		50	10000	10000	6030	16030	18610 (18611.5)	(1.16)	
reactive yellow K-4G	EPIDMA-bentonite	95		50	10000	12000	5850	17850	22060 (22056.4)	(1.24)	
reactive yellow K-4G	EPIDMA-bentonite	99		50	10000	15000	5520	20520	30070 (30072.8)	(1.47)	
disperse yellow brown S-2RFL	EPIDMA-bentonite	85	Langmuir isotherm: *q*_m_ = 12.36 mg/g, *K*_L_= 0.06 L/mg; *C*_e_: mg/L and *q*_e_: mg/g	50	10000	50000	27250	77250	110800 (110796)	(1.43)	(81)
disperse yellow brown S-2RFL	EPIDMA-bentonite	90		50	10000	60000	34860	94860	157770 (157766)	(1.66)	
disperse yellow brown S-2RFL	EPIDMA--bentonite	95		50	10000	80000	52110	132110	294630 (294633)	(2.23)	
disperse yellow brown S-2RFL	EPIDMA--bentonite	99		50	10000	160000	122800	282800	1375000 (1375000)	(4.86)	
atrazine	RSBC	95	Freundlich isotherm: *K*_f_ = 1680 mg^1–*n*^ L^*n*^/kg, *n* = 0.64	10	1000	900	1540	2440	8840 (8812)	(3.61)	(82)
atrazine	T-RSBC	95	Freundlich isotherm: *K*_f_ = 2716 mg^1–*n*^ L^*n*^/kg, *n* = 0.46	10	1000	650	770	1420	4470 (4811)	(3.39)	(82)
imidacloprid	RSBC	95	Freundlich isotherm: *K*_f_ = 3410 mg^1–*n*^ L^*n*^/kg, *n* = 0.51	10	1000	500	720	1220	3970 (3967)	(3.25)	(82)
imidacloprid	T-RSBC	95	Freundlich isotherm: *K*_f_ = 3140 mg^1–*n*^ L^*n*^/kg, *n* = 0.40	10	1000	500	880	1380	3980 (3992)	(2.89)	(82)
atrazine	RSBC	90	Freundlich isotherm: *K*_f_ = 1680 mg^1–*n*^ L^*n*^/kg, *n* = 0.64	10	1000	750	970	1720	5360 (5357)	(3.11)	(82)
atrazine	T-RSBC	90	Freundlich isotherm: *K*_f_ = 2716 mg^1–*n*^ L^*n*^/kg, *n* = 0.46	10	1000	500	590	1090	3050 (3313.6)	(3.04)	(82)
imidacloprid	RSBC	90	Freundlich isotherm: *K*_f_ = 3410 mg^1–*n*^ L^*n*^/kg, *n* = 0.51	10	1000	500	430	930	2640 (2639.2)	(2.84)	(82)
imidacloprid	T-RSBC	90	Freundlich isotherm: *K*_f_ = 3140 mg^1–*n*^ L^*n*^/kg, *n* = 0.40	10	1000	500	590	1090	2870 (2866.2)	(2.63)	(82)
simazine	Al_2_O_3_	95	Freundlich isotherm: *K*_f_ = 168 μmol kg^–1^, *n* = 0.444	20 μmol/L	1000	3840	6340	10180	113000 (113100)	(11.1)	(83)
simazine	Fe_2_O_3_	95	Freundlich isotherm: *K*_f_ = 166 μmol kg^–1^, *n* = 0.596	20 μmol/L	1000	5750	7820	13570	114000 (114460)	(8.4)	(83)
MCPA^f^	Al_2_O_3_	95	Langmuir isotherm: *q*_m_ = 2.01 × 10^5^ μmol kg^–1^, *K*_L_ = 5.05 × 10^–3^ L μmol^–1^	20 μmol/L	1000	1190	1230	2420	12290 (18810)	(5.08)	(83)
MCPA^f^	Fe_2_O_3_	95	Langmuir isotherm: *q*_m_ = 5.42 × 10^4^ μmol kg^–1^, *K*_L_ = 0.452 L μmol^–1^	20 μmol/L	1000	1800	1710	3510	16200 (16240)	(4.62)	(83)
zinc from effluents	sodium diimidoacetate ion exchange resin	99	Langmuir–Freundlich isotherm: *K*_LF_ = 6007.78 L/g, *a*_LF_ = 3572.5813 L/mmol, *b*_LF_ = 0.9884; *C*_e_: mmol/L and *q*_e_: mmol/g	1.7 mmol/dm^3^	1.7	1.475	0.2359	1.71	(1.73)	(1.01)	(74)
zinc from effluents	sodium diimidoacetate ion exchange resin	99		2 mmol/dm^3^	1.7	1.7339	0.2761	2.01	(2.02)	(1)	
zinc from effluents	sodium diimidoacetate ion exchange resin	99		2.3 mmol/dm^3^	1.7	1.9928	0.3163	2.31	(2.33)	(1.01)	
zinc from effluents	sodium diimidoacetate ion exchange resin	99		2.6 mmol/dm^3^	1.7	2.2517	0.3564	2.61	(2.63)	(1.01)	
zinc from effluents	sodium diimidoacetate ion exchange resin	99		2.9 mmol/dm^3^	1.7	2.5105	0.3965	2.91	(2.93)	(1.01)	
zinc from effluents	sodium diimidoacetate ion exchange resin	95		1.7 mmol/dm^3^	1.7	1.1484	0.4846	1.63	(1.64)	(1.01)	
zinc from effluents	sodium diimidoacetate ion exchange resin	95		2 mmol/dm^3^	1.7	1.3505	0.5692	1.92	(1.93)	(1.01)	
zinc from effluents	sodium diimidoacetate ion exchange resin	95		2.3 mmol/dm^3^	1.7	1.5527	0.6537	2.21	(2.21)	(1)	
zinc from effluents	sodium diimidoacetate ion exchange resin	95		2.6 mmol/dm^3^	1.7	1.7547	0.7382	2.49	(2.50)	(1)	
zinc from effluents	sodium diimidoacetate ion exchange resin	95		2.9 mmol/dm^3^	1.7	1.9567	0.8227	2.78	(2.8)	(1)	
zinc from effluents	sodium diimidoacetate ion exchange resin	90		1.7 mmol/dm^3^	1.7	0.8638	0.6809	1.54	(1.55)	(1.01)	
zinc from effluents	sodium diimidoacetate ion exchange resin	90		2 mmol/dm^3^	1.7	1.0158	0.8004	1.82	(1.82)	(1)	
zinc from effluents	sodium diimidoacetate ion exchange resin	90		2.3 mmol/dm^3^	1.7	1.168	0.9196	2.09	(2.1)	(1)	
zinc from effluents	sodium diimidoacetate ion exchange resin	90		2.6 mmol/dm^3^	1.7	1.32	1.0391	2.36	(2.36)	(1)	
zinc from effluents	sodium diimidoacetate ion exchange resin	90		2.9 mmol/dm^3^	1.7	1.4722	1.1582	2.63	(2.64)	(1)	

a If *q* is greater than the theoretically obtained *q*_e_, then removal is not possible in a single-stage adsorption unit.

b *C*_0_, initial concentration; *V*, volume; *M*_1_, adsorbent mass required to remove a fixed percentage of solute in stage 1 of a two-stage adsorption unit at equilibrium; *M*_2_, adsorbent mass required to remove a fixed percentage of solute in stage 2 of a two-stage adsorption unit at equilibrium; *M*_1_ + *M*_2_, total adsorbent mass required to remove a fixed percentage of solute in a two-stage adsorption unit); and *M*, adsorbent mass required to remove a fixed percentage of solute from the bulk liquid using a single-stage adsorption unit at equilibrium.

c Unless specified, initial concentration is expressed in mg/L.

d All the values in parentheses are calculated by us using the procedures explained insection 3.

e Polyepichlorohydrin-dimethylamine (EPIDMA) cationic polymer modified bentonite.

f 2-Methyl-4-chlorophenoxyacetic acid.

## 6 Contact Time Optimization Using Theoretical Adsorption Kinetics: Review

Table 5 shows the different combinations of the adsorbent/adsorbate systems and the optimum contact times required for a fixed a percentage removal of adsorbate from the bulk liquid. For the purpose of a comparison, we also show the contact times required to remove the same percentage of solute using a single-stage adsorption unit. Table 5 also presents the theoretical adsorption kinetic expressions used to optimize the contact time. Clearly, for a fixed percentage removal of solution, adsorption carried out in two stages significantly minimizes the contact time when compared with the amount of time required to remove the same percentage of solute using a single-stage adsorption unit. For all the systems detailed in Table 5, the ratio of the contact time required to remove a fixed percentage of some solute using a two-stage versus a single-stage adsorption unit is shown. Depending on the type of adsorption system and the initial concentration, the adsorption contact time required to remove a fixed percentage removal of solute in the two-stage sorption system was roughly 2–5 times lower than that using a single-stage adsorption unit.

**Table 5:** Contact Time Optimized Using Different Theoretical Adsorption Kinetics for a Wide Range of Adsorbents/Adsorbates^a^^,^^b^^,^^c^

adsorbate	adsorbent	% removal	theoretical kinetics	*C*_0_^d^ (mg/L)	*V* (L)	*M* (g)	*t*_1_ (min)	*t*_2_ (min)	*t*_1_ + *t*_2_ (min)	*t* (min)	*t*/(*t*_1_ + *t*_2_)^e^	ref
MCB^f^	pine sawdust	90	pseudo second order: *k* = 2.72 × 10^–3^ g/mg·min, *q*_e_ = 83.3 mg/g	150	10000	15000	20	25.9	45.9	(NR^g^)	(NR^g^)	(84)
MCB	pine sawdust	85		150	10000	15000	14	16.4	30.4	(NR^g^)	(NR^g^)	
MCB	pine sawdust	80		150	10000	15000	10	12.3	22.3	600 (107)	(3.52)	
MCB	pine sawdust	75		150	10000	15000	8	9.2	17.2	75 (40)	(2.32)	
MCY^h^	pine sawdust	90	pseudo second order: *k* = 1.29 × 10^–3^ g/mg·min, *q*_e_ = 108.7 mg/g	150	10000	15000	10	12.4	22.4	38 (34)	(1.52)	(84)
MCY	pine sawdust	85		150	10000	15000	8	9.6	17.6	27 (26)	(1.48)	
MCY	pine sawdust	80		150	10000	15000	6	8.3	14.3	21 (20)	(1.4)	
MCY	pine sawdust	75		150	10000	15000	6	5.8	11.8	16 (16)	1.36	
acid red 25	magnetic biomass	96	pseudo second order: *k* = 7.1 × 10^–3^ g/mg·min, *q*_e_ = 598.6 mg/g	150	10000	5000	180	220.8	400.8	895 (3^d^)	2.23^e^ (*t* ≪ *t*_1_ + *t*_2_)	(85)
acid red 25	magnetic biomass	90		150	10000	5000	140	143.9	283.9	599 (2^d^)	2.1^e^ (*t* ≪ *t*_1_ + *t*_2_)	
acid red 25	magnetic biomass	86		150	10000	5000	120	109.5	229.5	476 (2^d^)	2.1^e^ (*t* ≪ *t*_1_ + *t*_2_)	
acid red 25	magnetic biomass	83		150	10000	5000	100	92.6	192.6	356 (2^d^)	1.84^e^ (*t* ≪ *t*_1_ + *t*_2_)	
acid red 25	magnetic biomass	80		150	10000	5000	100	62.6	162.6	376 (2^d^)	2.31^e^ (*t* ≪ *t*_1_ + *t*_2_)	
methylene blue	sawdust	99	pseudo second order: *k* = 4591.5*C*_0_^–2.555^ g/mg·min, *q*_e_ = 0.2328*C*_0_^1.0142^ mg/g	300	1	4	26	11.54	37.54	(308)	(8.19)	(86)
methylene blue	sawdust	99		350	1	4	32	14.37	46.37	(351)	(7.56)	
methylene blue	sawdust	99		400	1	4	42	14.18	56.18	(396)	(7.05)	
methylene blue	sawdust	99		450	1	4	44	21.55	65.55	(443)	(6.76)	
methylene blue	sawdust	99		500	1	4	52	23.79	75.79	(492)	(6.49)	
methylene blue	sawdust	98		300	1	4	16	12.02	28.02	(202)	(7.21)	
methylene blue	sawdust	98		350	1	4	22	12.36	34.36	(238)	(6.94)	
methylene blue	sawdust	98		400	1	4	28	13.55	41.55	(276.3)	(6.65)	
methylene blue	sawdust	98		450	1	4	32	17.21	49.21	(316)	(6.41)	
methylene blue	sawdust	98		500	1	4	38	19.23	57.23	(355.9)	(6.22)	
methylene blue	sawdust	97		300	1	4	14	9.28	23.28	(149.6)	(6.43)	
methylene blue	sawdust	97		350	1	4	20	9	29	(179.8)	(6.2)	
methylene blue	sawdust	97		400	1	4	24	11.2	35.2	(211.2)	(6)	
methylene blue	sawdust	97		450	1	4	28	13.75	41.75	(243.8)	(5.84)	
methylene blue	sawdust	97		500	1	4	32	16.65	48.65	(277.5)	(5.7)	
Pb^2+^ ions from wastewater	NTB-modified kaolinite clay^i^	95	pseudo second order: *k* = 1.366 × 10^–2^ g/mg·min, *q*_e_ = 22.18 mg/g	200	10000	5000	12	27.7	39.7	(NR^g^)	(NR^g^)	(87)
Pb^2+^ ions from wastewater	NTB-modified kaolinite clay	90		200	10000	5000	10	28.1	38.1	(NR^g^)	(NR^g^)	
Pb^2+^ ions from wastewater	NTB-modified kaolinite clay	85		200	10000	5000	10	26.6	36.6	(NR^g^)	(NR^g^)	
Pb^2+^ ions from wastewater	NTB-modified kaolinite clay	80		200	10000	5000	10	25	35	(NR^g^)	(NR^g^)	
Cd^2+^ ions from wastewater	NTB-modified kaolinite clay	95	pseudo second order: *k* = 2.31 × 10^–2^ g/mg·min, *q*_e_ = 25.212 mg/g	200	10000	5000	4	9.9	13.9	(NR^g^)	(NR^g^)	(87)
Cd^2+^ ions from wastewater	NTB-modified kaolinite clay	90		200	10000	5000	4	9.4	13.4	(NR^g^)	(NR^g^)	
Cd^2+^ ions from wastewater	NTB-modified kaolinite clay	85		200	10000	5000	4	8.9	12.9	(NR^g^)	(NR^g^)	
Cd^2+^ ions from wastewater	NTB-modified kaolinite clay	80		200	10000	5000	4	8.4	12.4	(NR^g^)	(NR^g^)	
methylene blue	spent tea leaves	99	pseudo second order: *k* = 162.2*C*_0_^–1.88^ g/mg·min, *q*_e_ = 0.282*C*_0_^–1.002^ mg/g	300	0.5	1.75	18	10.1	28.1	(388.9)	(13.84)	(88)
methylene blue	spent tea leaves	99		400	0.5	1.75	22	13.9	35.9	(468.4)	(13.05)	
methylene blue	spent tea leaves	99		500	0.5	1.75	26	17.5	43.5	(542.6)	(12.47)	
methylene blue	bentonite	99	pseudo second order: *k* = 13.09 × 10^–4^ g/mg·min, *q*_e_ = 103.1 mg/g	100	10000	10000	40	64.6	104.6	(178.9)	(1.71)	(89)
methylene blue	bentonite	95		100	10000	10000	22	25.6	47.6	(86.9)	(1.83)	
methylene blue	bentonite	90		100	10000	10000	14	16.7	30.7	(50.9)	(1.66)	
methylene blue	bentonite	85		100	10000	10000	12	10	22	(34.8)	(1.58)	
basic blue 69	peat	90	pseudo second order: *k* = 1.84 × 10^–3^ g/mg·min, *q*_e_ = 86.4 mg/g	200	5000	10000	7.03	20.06	27.1	(NR^g^)	(NR^g^)	(20)
basic blue 69	wood	80	pseudo second order: *k* = 5.58 × 10^–4^ g/mg·min, *q*_e_ = 62.2 mg/g	200	5000	10000	89.75	77.20	166.9	(NR^g^)	(NR^g^)	(20)
acid blue 25	peat	80	pseudo second order: *k* = 7.47 × 10^–4^ g/mg·min, *q*_e_ = 12.7 mg/g	200	5000	10000	99.73	90.32	190.1	(NR^g^)	(NR^g^)	(20)
acid blue 25	wood	50	pseudo second order: *k* = 3.21 × 10^–3^ g/mg·min, *q*_e_ = 7.53 mg/g	200	5000	10000	79.6	89.7	169.4	(NR^g^)	(NR^g^)	(20)
phosphates	alunite	95	pseudo second order: *k* = 1.31 × 10^–3^ g/mg·min, *q*_e_ = 87.7 mg/g	100	10000	10000	44	45.7	89.7	(NR^g^)	(NR^g^)	(90)
phosphates	alunite	90		100	10000	10000	30	24.1	54.1	(NR^g^)	(NR^g^)	
phosphates	alunite	85		100	10000	10000	18	20	38.0	600 (274)	(7.21)	
phosphates	alunite	80		100	10000	10000	14	15.1	29.1	170 (90)	(3.1)	
methylene blue	coconut shell activated carbon	98.8	pseudo second order: *k* = 0.012 g/mg·min, *q*_e_ = 11.24 mg/g	100	0.025	0.25	6.64	0.08	6.7	(53.8)	(8.01)	(91)
Pb^2+^ ions from wastewater	polyvinyl-modified kaolinite clay	95	pseudo second order: *k* = 5.96*C*_0_^–1.0601^ g/mg·min, *q*_e_ = 0.4592*C*_0_^0.7765^ mg/g	300	2500	2000	6	24.2	30.2	(NR^g^)	(NR^g^)	(92)
Pb^2+^ ions from wastewater	polyvinyl-modified kaolinite clay	90		300	2500	2000	6	22.1	28.1	(NR^g^)	(NR^g^)	
Pb^2+^ ions from wastewater	polyvinyl-modified kaolinite clay	85		300	2500	2000	6	21.6	27.6	(NR^g^)	(NR^g^)	
Pb^2+^ ions from wastewater	polyvinyl-modified kaolinite clay	80		300	2500	2000	6	20.4	26.4	(NR^g^)	(NR^g^)	
Pb^2+^ ions from wastewater	tripolyphosphate-modified kaolinite clay	95	pseudo second order: *k* = 1.01 × 10^–2^ ± 0.0036 g/mg·min, *q*_e_ = 34.96 ± 1.57 mg/g	500	2500	2000	8	26.3	34.3	(NR^g^)	(NR^g^)	(93)
Pb^2+^ ions from wastewater	tripolyphosphate-modified kaolinite clay	80		500	2500	2000	8	22.2	30.2	(NR^g^)	(NR^g^)	
Pb^2+^ ions from wastewater	tripolyphosphate-modified kaolinite clay	70		500	2500	2000	6	21.2	27.2	(NR^g^)	(NR^g^)	
Pb^2+^ ions from wastewater	tripolyphosphate-modified kaolinite clay	50		500	2500	2000	6	15.1	21.1	(NR^g^)	(NR^g^)	
MCPA	mesoporous Al_2_O_3_	95.4	pseudo second order: *k* = 0.129 × 10^–6^ μmol/min, *q*_e_ = 8.92 × 10^5^ μmol/kg	150 μmol/L	0.02	0.02	1396	655	2051	(1660.58)	(0.84)	(94)
MCPA	mesoporous Al_2_O_3_	95		150 μmol/L	0.02	0.02	920	437	1357	(1652.29)	(1.22)	
MCPA	mesoporous Al_2_O_3_	94		150 μmol/L	0.02	0.02	498	243	741	(1631.6)	(2.2)	
MCPA	mesoporous Al_2_O_3_	93		150 μmol/L	0.02	0.02	342	171	513	(1611.06)	(3.14)	
MCPA	mesoporous Al_2_O_3_	92		150 μmol/L	0.02	0.02	261	132	393	(1590.57)	(4.05)	
MCPA	mesoporous Al_2_O_3_	91		150 μmol/L	0.02	0.02	210	110	320	(1570.16)	(4.91)	
MCPA	mesoporous Al_2_O_3_	90		150 μmol/L	0.02	0.02	177	93	270	(1549.82)	(5.74)	
MCPA	mesoporous Al_2_O_3_	85		150 μmol/L	0.02	0.02	97	56	153	(1449.36)	(9.47)	
MCPA	mesoporous Al_2_O_3_	80		150 μmol/L	0.02	0.02	66	41	107	(1350.85)	(12.62)	
MCPA	mesoporous Al_2_O_3_	75		150 μmol/L	0.02	0.02	49	32	81	(1254.24)	(15.48)	
MCPA	mesoporous Al_2_O_3_	70		150 μmol/L	0.02	0.02	39	26	65	(1159.47)	(17.84)	
MCPA	mesoporous Al_2_O_3_	65		150 μmol/L	0.02	0.02	31	22	53	(1066.48)	(20.12)	
MCPA	mesoporous Al_2_O_3_	60		150 μmol/L	0.02	0.02	26	18	44	(975.24)	(22.16)	
MCPA	mesoporous Al_2_O_3_	55		150 μmol/L	0.02	0.02	21	16	37	(885.69)	(23.94)	
MCPA	mesoporous Al_2_O_3_	50		150 μmol/L	0.02	0.02	18	13	31	(797.78)	(25.73)	
Pb^2+^ ions	peat	99	pseudo second order: *k* = 1.45 × 10^4^*C*_0_^–2.72^ g/mg·min, *q*_e_ = 0.487*C*_0_^0.087^ mg/g	400	5000	20000	22	11.9	33.9	(NR^g^)	(NR^g^)	(21)
Pb^2+^ ions	peat	98		400	5000	20000	10	45.8	55.8	(1640.79)	(29.4)	
Pb^2+^ ions	peat	96		400	5000	20000	18	8.06	26.06	(321.46)	(12.34)	
Pb^2+^ ions	peat	94		400	5000	20000	14	8.98	22.98	(174.87)	(7.61)	
Pb^2+^ from aqueous solution	modified sugar cane bagasse	90	pseudo second order: *k* = 6 × 10^–4^ g/mg·min, *q*_e_ = 18.3 mg/g	40	0.05	0.1	14	13.84	27.84	(5464.5)	(196.2)	(95)
Pb^2+^ from aqueous solution	modified sugar cane bagasse	95		40	0.05	0.1	22	19.61	41.61	(NR^g^)	(NR^g^)	
Pb^2+^ from aqueous solution	modified sugar cane bagasse	98		40	0.05	0.1	34	32.27	66.27	(NR^g^)	(NR^g^)	
zinc from effluents	sodium diimidoacetate ion exchange resin	90	pseudo second order: *k* = 0.007*C*_0_^0.393^ g/mg·min, *q*_e_ = 2.40*C*_0_^–0.13^ mg/g	2 mmol/dm^3^	1.7	1.1	91.5	91.5	183	(NR^g^)	(NR^g^)	(74)
zinc from effluents	sodium diimidoacetate ion exchange resin	95		2 mmol/dm^3^	1.7	1.1	101.3	110.8	212.1	(NR^g^)	(NR^g^)	
zinc from effluents	sodium diimidoacetate ion exchange resin	90		2.3 mmol/dm^3^	1.7	1.1	140.1	142.7	282.8	(NR^g^)	(NR^g^)	
zinc from effluents	sodium diimidoacetate ion exchange resin	95		2.3 mmol/dm^3^	1.7	1.1	170.8	175.5	346.3	(NR^g^)	(NR^g^)	
zinc from effluents	sodium diimidoacetate ion exchange resin	90		2.6 mmol/dm^3^	1.7	1.1	249.3	260.8	510.1	(NR^g^)	(NR^g^)	
zinc from effluents	sodium diimidoacetate ion exchange resin	95		2.6 mmol/dm^3^	1.7	1.1	100.8	110.9	211.7	(NR^g^)	(NR^g^)	

a *C*_0_, initial concentration; *V*, volume; *M*, adsorbent mass; *t*_1_, time required to remove a fixed percentage of solute in stage 1 of a two-stage adsorption unit; *t*_2_, time required to remove a fixed percentage of solute in stage 2 of a two-stage adsorption unit; *t*_1_ + *t*_2_, time required to remove a fixed percentage of solute in a two-stage adsorption unit; and *t*, time required to remove a fixed percentage of solute using a single-stage adsorption unit.

b The units of the isotherm constants given in this table were reported based on the units reported in the literature cited.

c All the values in the parentheses are calculated by us using the procedures explained in section 4.

d Unless specified, initial concentration is expressed in terms of mg/L.

e *t*/(*t*_1_ + *t*_2_) is obtained based on the values reported in the literature; however, according to our calculated values, *t* ≪ *t*/(*t*_1_ + *t*_2_).

f Metal complex blue.

g NR, removal is not possible in a single-stage adsorption unit.

h Metal complex yellow.

i NTB, sodium tetraborate.

Clearly, removal of solute using a multistage adsorption unit minimizes the contact time required to achieve a fixed percentage removal of solute from bulk solutions. In terms of the theoretical adsorption kinetics used to optimize the contact time, at least based on the adsorbent/adsorbate systems reviewed in Table 5, it is clear that the pseudo-second-order expression is the most widely used expression to minimize the contact time. Benefits of this kinetic expression are that it is simple to use, the kinetic parameters can be obtained using a simple regression analysis, and, most importantly, it represents the most of the experimental adsorption kinetic data well (irrespective of the type of adsorbent and adsorbate). In addition to the works reported in Table 5, a few researchers used other kinetic models like Vermeulen’s approximation and the pore-diffusion model. It must be stressed here that the only required information to optimize the adsorption contact time is the kinetic parameters that can essentially capture the change in the amount adsorbed as a function of time at different operating conditions. Thus, it is essential to identify a suitable theoretical adsorption kinetics that well represent the experimental adsorption kinetic data. Once a suitable theoretical adsorption kinetics is identified, then mathematically, irrespective of the type of the theoretical kinetic expression used, the contact time can be optimized using the procedures discussed in section 4.

## 7 Concluding Remarks

In this work, we reviewed literature that report on the design of adsorption systems with a target to minimize either the contact time or the adsorbent loading. On the basis of our review, we realized that the design calculations reported in the literature indicate that adsorption contact time and adsorbent loading can be minimized by simply performing the batch adsorption experiments in multiple stages. This means more volumes of solution can be treated effectively in less time and with less adsorbent. To complement the review, we provided model calculations that will help to optimize adsorbent mass and contact time using a multistage adsorption unit. For the convenience of the readers, all the calculations performed or shown in this review are detailed in Microsoft Excel spreadsheets and made available to the readers as Supporting Information. In the Supporting Information, we show how to obtain the isotherm parameters using linear and nonlinear regression analyses, optimize the contact mass using a model theoretical adsorption isotherm, and optimize contact time using theoretical adsorption kinetics. In the model calculations we used the established Langmuir adsorption isotherms and pseudo-second-order kinetics. The sample calculations can be easily adapted and modified to use other theoretical adsorption isotherms (most of the theoretical adsorption isotherms are presented in Table 1) and kinetics to optimize the adsorbent mass and the contact time, respectively. The design procedures reviewed and discussed in this work are extremely simple to perform and rely only on theoretical adsorption isotherms, kinetics, and mass balance expressions. If required, the design procedures discussed in this review work can be used to scale up the single-stage or multistage adsorption unit, and this can be done by simultaneously increasing the volume of the solution and the adsorbent loading.

The design methods and the calculation procedures explained in this review are universal and can be widely used to optimize the contact time and adsorbent loading during the removal of a wide range of solutes from their aqueous solutions using adsorption techniques. Currently, researchers are using different classes of porous materials, like metal–organic frameworks,^96^ covalent organic frameworks,^97^ zeolitic imidazolate frameworks,^98^ and graphene-based materials,^99^ to remove several pollutants from the aqueous phase. Most of these materials are relatively expensive when compared to the low-cost adsorbents or the carbon-based materials that are usually produced in large scale using chemical or physical activation techniques. Undoubtedly, optimization of the adsorbent mass and the contact time will minimize the overall cost of the process that relies on these expensive adsorbents. Finally, we would like to conclude that the design methods reviewed and discussed in this work will be beneficial when dealing with adsorption systems that rely on expensive adsorbents with slow adsorption kinetics or when large volumes of solution need to be processed.

## Supporting Information Available

Methods to obtain isotherm parameters using regression analysis (XLSX)
Methods to optimize adsorption contact time using theoretical adsorption kinetics (XLSX)
Methods to optimize the adsorbent mass using theoretical isotherms (XLSX)
